# Polarization-dependent angularly selective dielectric reflector

**DOI:** 10.1038/s41467-026-77529-6

**Published:** 2026-09-15

**Authors:** Hyosim Yang, Ki Young Lee, Sanghyeok Park, Jiho Noh, Kate Musick, Jaeyeon Yu, Tomás Santiago-Cruz, Chloe F. Doiron, Alexander Cerjan

**Affiliations:** 1 Center for Integrated Nanotechnologies, Sandia National Laboratories, Albuquerque, NM 87185 USA; 2 Sandia National Laboratories, Albuquerque, NM 87185 USA

**Keywords:** Photonic crystals, Metamaterials, Metamaterials

## Abstract

A key limitation of conventional polarizers is their lack of intrinsic angular selectivity. Thus, optical devices that manipulate polarization and propagation direction are forced to realize these two degrees of freedom sequentially, increasing size and complexity. Here, we introduce and experimentally demonstrate a compact, all-dielectric optical element that simultaneously achieves polarization separation and angular selectivity. This is accomplished using a truncated three-dimensional photonic crystal that possesses a mirror symmetry and exhibits an associated symmetry band gap. For incident light aligned with the high-symmetry direction, the symmetry band gap enforces polarization-dependent reflection and transmission, whereas deviations from this orientation lift the symmetry constraint and erase the polarization selectivity. Implemented using alternating layers of silicon-rich and silicon-poor nitride etched into a cubic lattice of prisms, the structure exhibits tailorable bandwidth and low optical loss while maintaining stable performance over a wide field of view and preserving compatibility with standard nanofabrication techniques.

## Abstract (web summary)

A symmetry band gap in a three-dimensional photonic crystal enables simultaneous control of light’s polarization and propagation direction, yielding a compact, broadband, low-loss dielectric optical element with strong angular selectivity.

## Introduction

Polarization control provides key functionalities across diverse application domains such as imaging^1,2^, optical information processing^3^, displays^4^, and sensing^5,6^, by enabling contrast enhancement^7^, information separation^8^, and improved signal selectivity^9^. Recently, its importance has expanded with the development of emerging technologies such as augmented and virtual reality (AR/VR)^10^, computational imaging^11^, polarization-controlled holography^12^, and ultrafast detection and modulation^13^. Conventional polarization control elements including birefringent components^14^, wire grids^15^, and nanoparticle-based devices^16^, typically rely on polarization-dependent phase retardation, reflection or absorption. While such polarization optics can efficiently separate orthogonal polarization states, they treat polarization as a global property of the optical field and do not inherently provide angularly selective responses. As a result, applications requiring simultaneous control over polarization and propagation direction realize these two functionalities independently, achieving directional control through additional optical stages such as real-space spatial filtering or Fourier-plane **k**-space filtering^17–19^. Unfortunately, these additional optical elements needed to simultaneously control polarization and propagation direction increase system complexity and spatial footprint, ultimately leading to greater cumulative optical losses.

Achieving dual control over polarization and propagation direction would have a substantial impact in optical technologies where size and weight are at a premium. For example, in wearable AR systems, virtual information must be selectively reflected toward the viewer’s eye, while the real-world scene should be transmitted with high fidelity and minimal loss^20–32^. Similarly, defect and edge detection in compact imaging systems^33,34^ can be realized through tailored angular optical responses to achieve analog feature extraction^19,35–37^, as the requisite convolution kernels correspond to Fourier-space filters via the convolution theorem. While prior metasurface, metamaterial, and photonic crystal–based approaches have demonstrated individual aspects of these capabilities, including wide-field polarization control, engineered angular responses, and compact analog image processing^38–52^, next-generation AR/VR and imaging applications highlight the need for optical systems in which polarization control, operational bandwidth, and angular selectivity emerge intrinsically in a single element, rather than being imposed by external architectures.

**Fig. 1:**
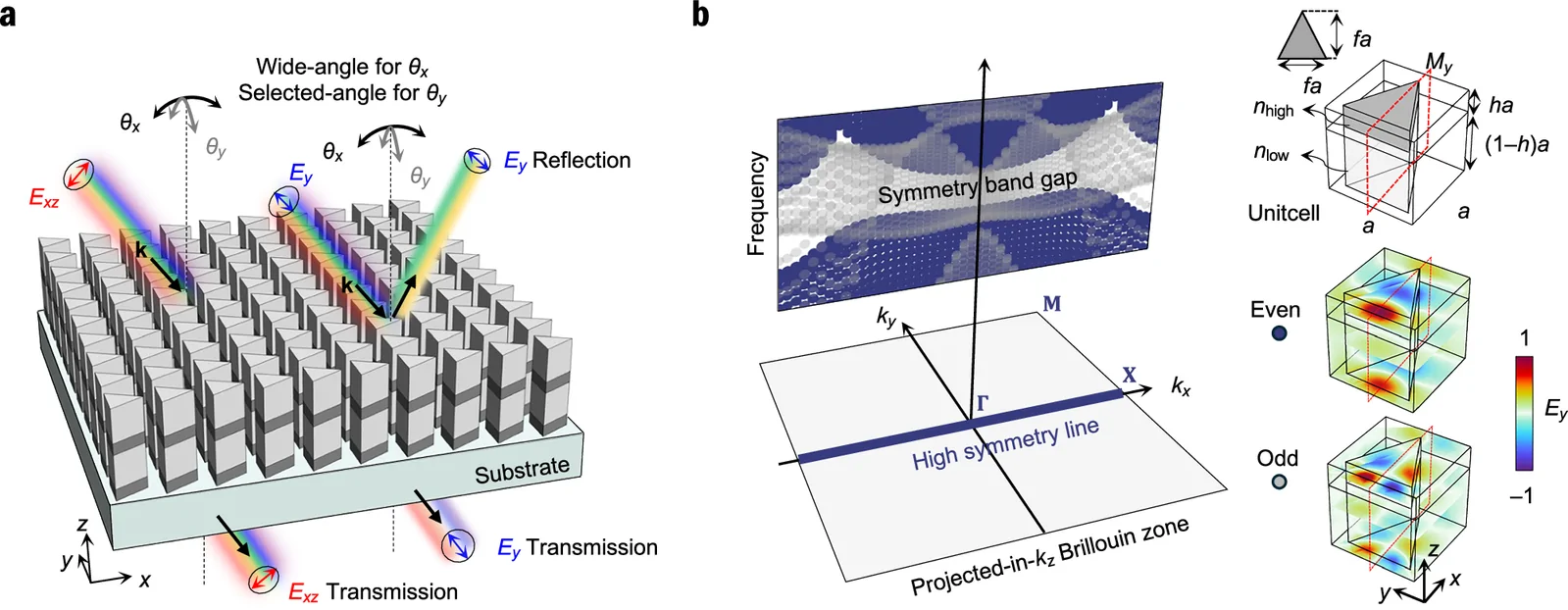
Angle- and polarization-selective dielectric reflector enabled by structural symmetry. **a** Schematic of an angle- and polarization-selective dielectric reflector illustrating polarization-dependent reflection and transmission for incident light aligned with the system’s symmetry, with varying *θ*_*x*_ at *θ*_*y*_ = 0. The polarization selectivity disappears for incidence with increasing ∣*θ*_*y*_∣. The rainbow-colored beams schematically represent broadband incident light and wavelength-selective reflection. Blue and red labels denote the *E*_*y*_ and *E*_*xz*_ polarization channels, respectively (i.e., the *E*_*x*_ = 0 and *E*_*y*_ = 0 polarizations), and **k** denotes the incident wavevector. **b** Projected-in-*k*_*z*_ symmetry band gap of a layered prism 3D PhC calculated along the high-symmetry line *k*_*y*_ = 0 that is formed from the mirror symmetry *M*_*y*_. The crystal’s Bloch modes are labeled by even (blue) and odd (gray) parity with respect to their *E*_*y*_ field components under the mirror operator *M*_*y*_. The unit-cell geometry is shown, defined by the lattice constant *a*, in-plane prism parameter *f*, and relative layer height *h*, alongside representative *E*_*y*_ electric-field distributions of the even and odd modes. The reflection symmetry plane of *M*_*y*_ is indicated in red. Simulations use *f* = 0.75, *h* = 0.25, *n*_high_ = 3.5, and *n*_low_ = 1.5.

Here, we introduce and experimentally demonstrate a thin, all-dielectric, angular- and polarization-selective optical element that operates over a tailorable spectral range and field of view, see Fig. 1a. The underlying mechanism of our element exploits mirror symmetries that can manifest in 3D photonic crystals (PhCs) to classify their Bloch modes into even- and odd-parity families along the corresponding high-symmetry planes of the crystal’s Brillouin zone. With proper tuning of the 3D PhC’s structure, it can achieve a symmetry band gap^53^, i.e., a spectral range along a high-symmetry plane in which the crystal’s Bloch eigenstates only exhibit a single parity character, not both. Thus, for frequencies within the symmetry band gap, a truncated version of such a 3D PhC exhibits polarization-dependent transmission and reflection for incident angles aligned with the high-symmetry direction, where the transmission of the polarization corresponding to the crystal’s missing parity character is strongly suppressed. When the incident angle deviates from this high-symmetry orientation, this missing parity character is rapidly restored and the polarization-selective response disappears, altogether resulting in pronounced angular- and polarization-dependence in the transmission and reflection spectra without relying on a resonant effect. We experimentally realize our optical element in an all-dielectric platform consisting of alternating layers of silicon-rich nitride (SRN) and silicon-poor nitride (SPN) patterned into a layered prism geometry to form a 3D PhC on a sapphire substrate. Our device exhibits strong polarization selectivity with normalized extinction ratios up to 14dB, corresponding to transmission below 6% (minimum 2%) for the blocked polarization in a truncated 3D PhC geometry that is only two unit cells tall, such that the structure is fabricated with a modest aspect ratio of 3:1 and a single vertical etch. The observed selectivity is maintained over an approximately 20nm spectral bandwidth and a single-axis field of view >50°, while this selectivity disappears for off-axis angle deviations ≥5°. Overall, our optical element demonstrates a compact, broadband, and low-loss platform for simultaneously controlling the polarization and propagation direction of light, offering a pathway toward integrated photonic systems with angular and polarization selectivity.

## Results

To illuminate how a compact polarization- and angular-selective optical element can be created using a 3D PhC, consider the band structure of a layered prism PhC formed from alternating, vertically stacked layers of two dielectric materials with different refractive indices. The combination of the triangular in-plane structural profile of the layered prisms with the square in-plane lattice periodicity results in the full 3D PhC possessing a mirror symmetry *M*_*y*_, such that *M*_*y*_(*x*, *y*, *z*) = (*x*, −*y*, *z*), see Fig. 1b. In wavevector space, this mirror symmetry acts as *M*_*y*_(*k*_*x*_, *k*_*y*_, *k*_*z*_) = (*k*_*x*_, −*k*_*y*_, *k*_*z*_), so the plane *k*_*y*_ = 0 is invariant under *M*_*y*_ and defines a high-symmetry plane. As such, for *k*_*y*_ = 0, the PhC’s Bloch eigenmodes can be classified through their spatial profiles as either even or odd with respect to the real-space mirror symmetry *M*_*y*_. By tailoring the relative thicknesses of the constituent material layers and the geometry of the layered prism motif, it is possible to find frequency ranges at *k*_*y*_ = 0 where only a single parity character exists in the 3D PhC, yielding a symmetry band gap^53^. For a layered prism PhC shown in Fig. 1b, a symmetry band gap generally opens in the even-in-*E*_*y*_ modes with *k*_*y*_ = 0 that can span the full range *k*_*x*_ ∈ [ −*π*/*a*, *π*/*a*) when the materials’ index contrast is sufficiently large, Δ*n* ≳ 0.8 for a fixed higher-index material with *n*_high_ = 3.5. Although the full 3D PhC remains gapless, the even- and odd-in-*E*_*y*_ modes with *k*_*y*_ = 0 belong to distinct *M*_*y*_ symmetry sectors with different field parity and nodal structures, so each symmetry sector can exhibit its own band gaps in certain frequency ranges. Note that it is sufficient to specify the symmetry of a single field component, as this uniquely determines the symmetry of all of the field components via Maxwell’s equations.

When the layered prism PhC is truncated to be semi-infinite in the vertical direction, the symmetry band gap strongly constrains the coupling of the free-space channels to the modes of the layered prism PhC. From the perspective of the layered prism PhC, the truncation breaks the system’s periodicity in *z*, such that *k*_*z*_ need not be conserved across the interface; this reduces the high-symmetry plane to a high-symmetry line in the 2D Brillouin zone. For frequencies within the symmetry band gap, only odd-in-*E*_*y*_ modes are available along the **Γ**–**X** high-symmetry line. Therefore, any even-in-*E*_*y*_ incident light has a vanishing symmetry overlap with these odd-in-*E*_*y*_ modes and cannot couple to a propagating mode inside the PhC, resulting in suppressed transmission. For incoming light with a wavevector in the *xz*-plane, the polarization condition *E*_*x*_ = 0 corresponds to an incident field with only an even-in-*E*_*y*_ electric-field component, independent of *θ*_*x*_ (see Fig. 1a), as the field profile of the incoming plane wave has no dependence on *y*. Note that for *k*_*y*_ = 0, the *E*_*x*_ = 0 polarization is the transverse-electric (TE), or *s*-polarized, free-space channel. Thus, TE incident light is expected to be suppressed within the symmetry band gap. In contrast, transverse-magnetic (TM), or *p*-polarized, incident light with *θ*_*y*_ = 0 satisfies *E*_*y*_ = 0, which corresponds to non-zero *E*_*xz*_ and is not symmetry-forbidden from coupling to the available odd-in-*E*_*y*_ modes, allowing transmission through the PhC. When the incident light is not aligned with the high-symmetry line, *θ*_*y*_ ≠ 0, the mirror symmetry constraint is lifted, and both incident polarizations can couple to the modes of the 3D PhC. In the following, we use the free-space polarization basis *E*_*x*_ = 0 and *E*_*y*_ = 0 to denote the two incident polarization channels for arbitrary incident angles (*θ*_*x*_, *θ*_*y*_), which maintains a straightforward connection to the separable symmetry sectors of the layered prism 3D PhC when *θ*_*y*_ = 0.

**Fig. 2:**
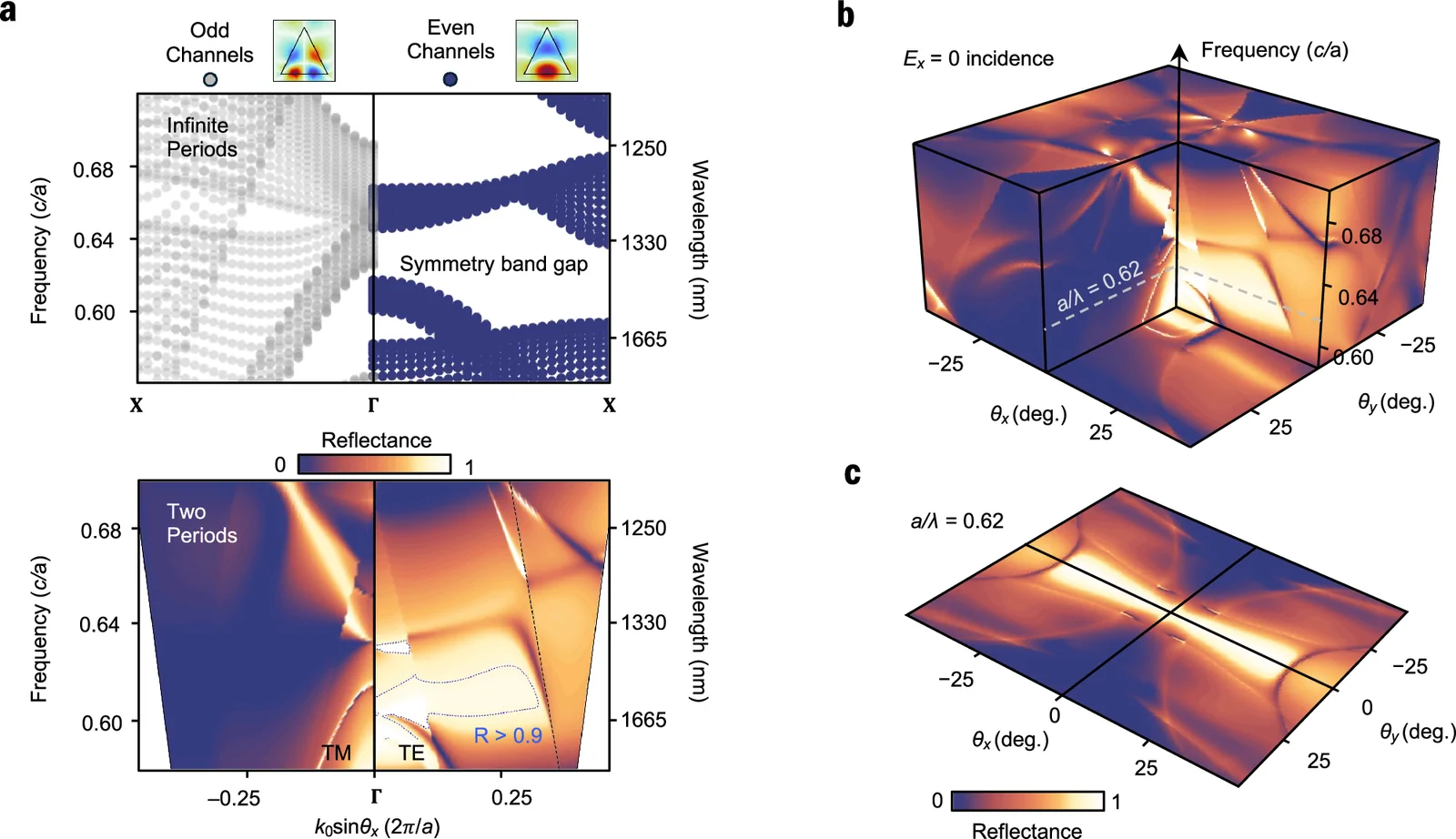
Connection between the polarization- and angular-selectivity in a two-period structure with the symmetry band gap of the infinite crystal. **a** Projected-in-*k*_*z*_ bands of the infinite layered-prism PhC along **Γ**–**X** separated by their even (blue) and odd (gray) *M*_*y*_ parity in their *E*_*y*_ field components, as well as the polarization-resolved reflectance of a two-unit-cell SRN/SPN structure at *θ*_*y*_ = 0. Blue and black dashed lines denote the contour where reflectance *R* = 0.9 and the light line, respectively. The layered prism structure is designed with *a* = 850nm, *f* = 0.415, *h* = 0.7, *n*_SRN_ = 2.9 and *n*_SPN_ = 1.8. **b** Angle- and frequency-resolved reflectance map of the two-period SRN/SPN structure for (*E*_*x*_ = 0)-polarized incident light. **c** Isofrequency reflectance within the symmetry band gap, showing strong angular dependence along the high-symmetry direction for (*E*_*x*_ = 0)-polarized incident light.

To demonstrate that the symmetry-governed angular and polarization selectivity predicted using an infinite structure can be preserved in a practical device with finite thickness, we compare the projected-in-*k*_*z*_ photonic bands of the even and odd parity modes along the **Γ**–**X** high-symmetry line with the polarization-dependent reflectance spectra of a two-unit-cell thick PhC in Fig. 2a. In particular, we consider a fabrication-feasible SRN/SPN structure operating in the near-infrared spectral range. We observe that the layered prism band structure possesses a complete symmetry band gap, manifesting as a high-reflectance window in the two-unit-cell structure spanning 1340–1400nm and incident angles up to *θ*_*x*_ ≈ 35°, as defined by the *R* = 0.9 contour in Fig. 2a under (*E*_*x*_ = 0)-polarized illumination at *θ*_*y*_ = 0. Likewise, the presence of odd-in-*E*_*y*_ modes over the same wavelength range in the infinite structure yields high transmission in the finite-height system for incident (*E*_*y*_ = 0)-polarized light. This polarization-dependent reflectivity is strongest below the Bragg-diffraction cutoffs, where the reflected light only has access to the specular reflection channel, see Methods for numerical details.

Off the high-symmetry-line, simulations of the (*E*_*x*_ = 0)-polarized reflection spectrum of a two-unit-cell-tall SRN/SPN prism structure confirm the angular selectivity of the finite structure due to the breakdown of the system’s mirror symmetry. In particular, the polarization-dependent reflectance remains extended along the *θ*_*x*_ direction, but disappears with increasing *θ*_*y*_ for any frequency within the symmetry band gap [Fig. 2b]. Similarly, an isofrequency reflectance map exhibits a rectangular angular response, elongated along *θ*_*x*_ and confined along *θ*_*y*_, indicating that high TE reflectance is localized along the symmetry-defined direction [Fig. 2c]. Overall, the observed agreement between the 3D PhC’s band structure and reflectance spectra of a finite variant of the system shows that the symmetry band gap formed by the unit-cell mirror symmetry simultaneously enforces polarization and angular selectivity, enabling broadband and wide-field-of-view operation along the high-symmetry orientation in a minimal two-period structure built from lossless dielectric materials without relying on system resonance. A more detailed explanation of the causal relation between a symmetry band gap and polarization-dependent electromagnetic scattering is provided in Supplementary Note 1, along with examples of additional structures that achieve this effect.

**Fig. 3:**
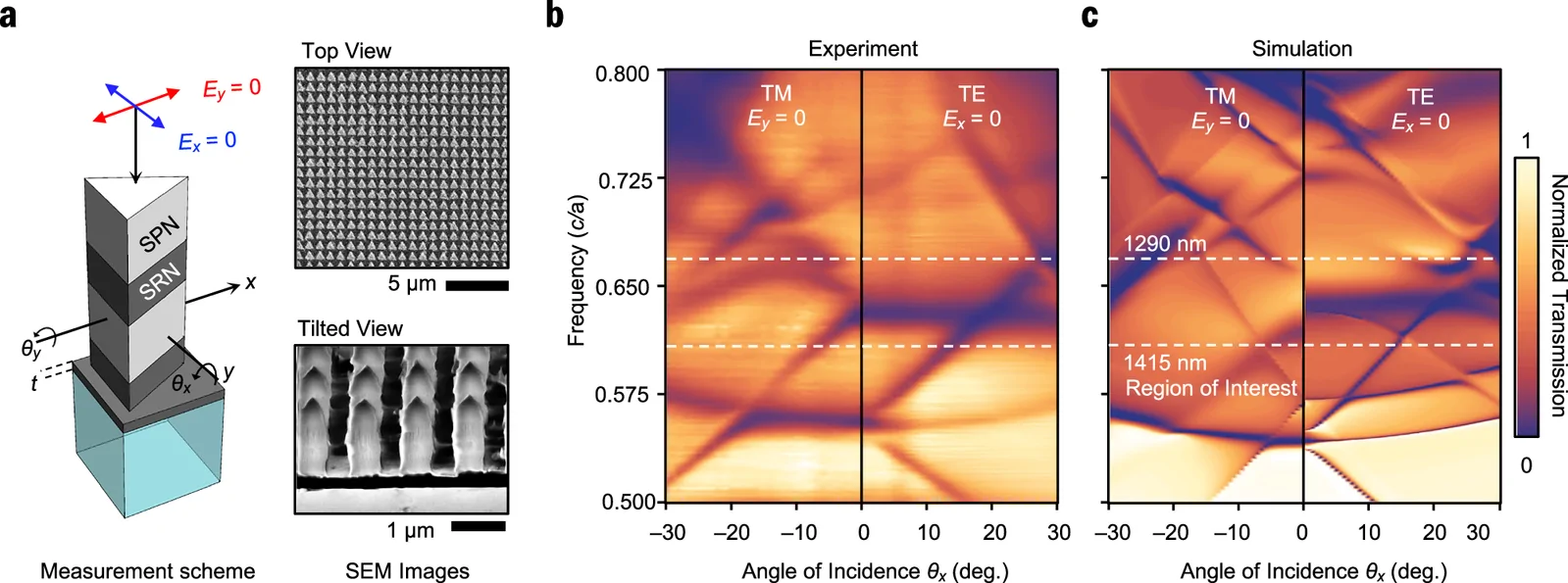
Experimental verification of polarization-selective transmission in the two-period layered prism structure. **a** Schematic of the optical measurement configuration, illustrating the rotation axes and incident polarization relative to the mirror symmetry of the layered-prism structure. Red and blue arrows denote the *E*_*y*_ = 0 (TM) and *E*_*x*_ = 0 (TE) polarization channels, respectively. The residual silicon-rich nitride (SRN) slab has thickness *t* = 100nm. Top- and tilted-view scanning electron microscopy (SEM) images of the fabricated triangular photonic-crystal array are shown on the right; SPN denotes silicon-poor nitride. **b** Experimental polarization-dependent normalized transmission as the sample is rotated from *θ*_*x*_ = −30° to 30° with *θ*_*y*_ = 0, corresponding to the symmetry-preserving plane. **c** Calculated polarization-dependent normalized transmission along the same symmetry-preserving orientation, incorporating fabrication-induced structural deviations. TE and TM denote transverse-electric and transverse-magnetic polarization, respectively. Colors in **b** and **c** represent normalized transmission from 0 to 1, as indicated by the color bar. The horizontal dashed lines in **b** and **c** mark wavelengths of 1290 and 1415 nm and bound the indicated region of interest.

We experimentally demonstrate a symmetry band gap–enabled polarization- and angular-selective reflector in a two-period SRN/SPN layered prism structure. The layers with approximate refractive indices *n*_SRN_ = 2.8 and *n*_SPN_ = 1.9 [see Supplementary Note 2] are deposited by plasma-enhanced chemical vapor deposition (PECVD) on a sapphire substrate, forming a four-layer stack. The in-plane triangular pillar array is patterned by electron-beam lithography, followed by a single dry etch step to form layered prisms with an aspect ratio of approximately 3:1 in height to in-plane prism edge length, see Methods for fabrication details. A schematic of the resulting layered prism geometry, together with top-view and tilted-view scanning electron microscope (SEM) images, is shown in Fig. 3a. The SEM images reveal a uniform triangular array over a large area, while the tilted-view SEM image shows that the bottom layer remains partially etched, leaving residual SRN approximately 100 nm thick. Despite this deviation from the desired geometry, the overall mirror symmetry of the unit cell remains intact.

To characterize the optical response of the fabricated samples, we perform angle-resolved transmission spectroscopy for both TE and TM polarized light. Using a broadband white light source, we scan the incident angle along the *θ*_*x*_ direction while maintaining *θ*_*y*_ = 0, and normalize all measured transmission spectra to those obtained from an unpatterned substrate measured under identical conditions. The incident polarization and angular configuration are illustrated in Fig. 3a, and further characterization details are provided in Supplementary Note 3.

Normalized transmission spectra of the two-period layered prism structure show a strong dependence on input polarization, with near-zero transmission over the wavelength range 1330 to 1350 nm under TE illumination aligned with the structure’s mirror symmetry, see Fig. 3b. At *λ*_c_ = 1340 nm, approximately at the center of the low-TE transmission wavelength range, normalized TE-polarized transmission is strongly suppressed *T*_TE_ ≤ 0.06 (minimum  ~0.02) for incident angles up to *θ*_*x*_ = ±25°. In contrast, normalized TM-polarized transmission remains above *T*_TM_ ≥ 0.5 over the same angular range, corresponding to polarization extinction ratios of up to 14dB, resulting in a wide field of view of polarization selective transmission when *θ*_*y*_ = 0. Simulations of our experimentally realized structure quantitatively reproduce the measured transmission spectra, see Fig. 3c, confirming that the suppressed-TE transmission feature originates in the symmetry band gap of the corresponding infinite layered prism 3D PhC.

**Fig. 4:**
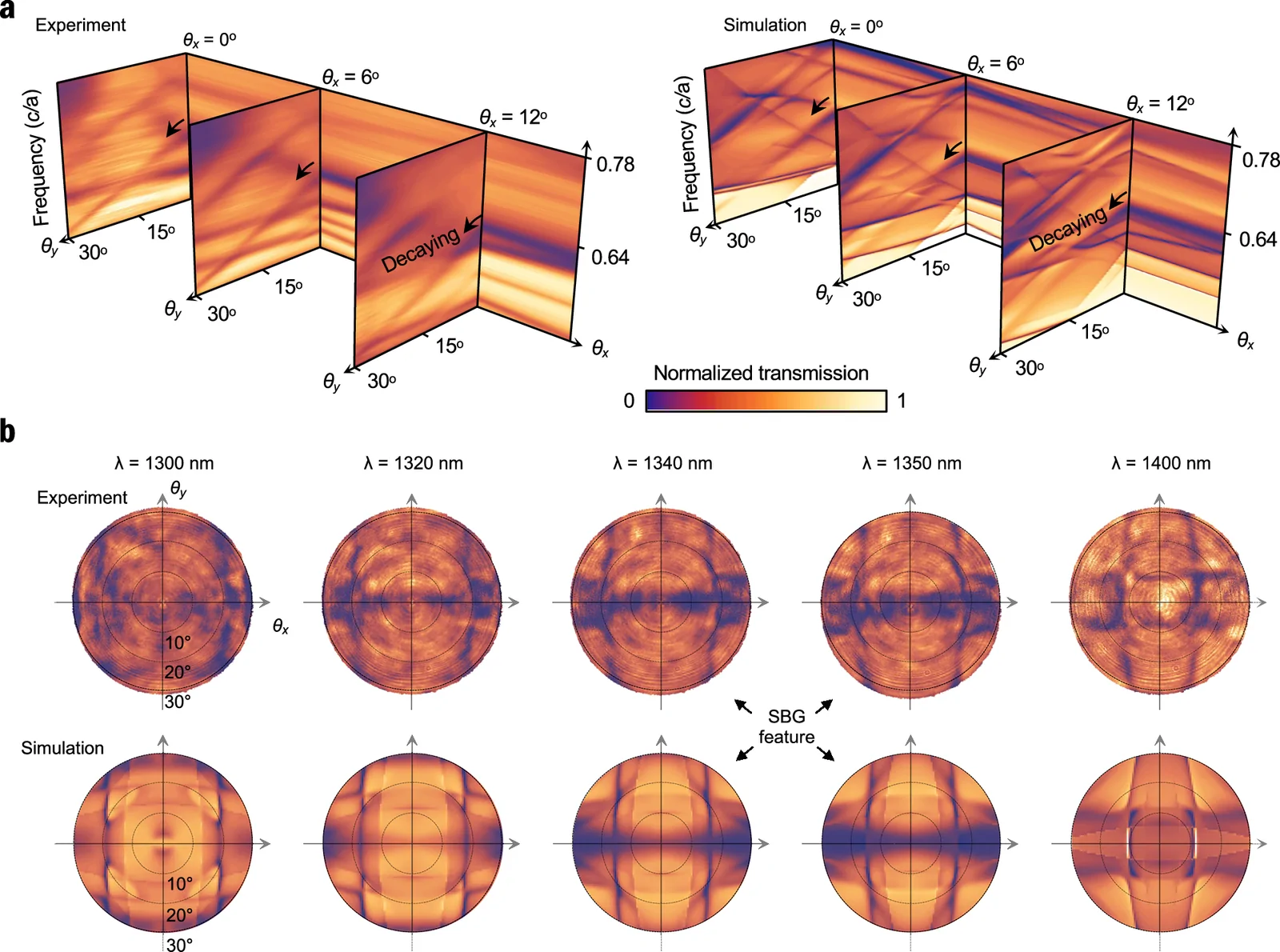
Experimental demonstration of intrinsic angular selectivity of the layered prism structure. **a** Experimental substrate-normalized transmission for *E*_*x*_ = 0 incident polarization as a function of *θ*_*y*_, obtained by varying the incident angle away from the high-symmetry orientation at fixed *θ*_*x*_ = 0^∘^, 6^∘^, and 12^∘^. The transmission is strongly suppressed only near *θ*_*y*_ = 0^∘^, corresponding to alignment with the symmetry-defined incident plane, and rapidly recovers as the incident angle deviates from this direction. Corresponding numerical simulations are shown on the right. Black arrows indicate the decay of the suppressed-transmission feature with increasing ∣*θ*_*y*_∣. **b** Experimental (top row) and simulated (bottom row) substrate-normalized back-focal-plane transmission maps for *E*_*x*_ = 0 incident polarization at representative wavelengths from *λ* = 1300 to 1400 nm. The dashed circles indicate polar angles of 10^∘^, 20^∘^, and 30^∘^. Colors in all panels represent substrate-normalized transmission from 0 to 1 using the common color scale shown in **a**. The symmetry band gap (SBG) feature appears within the measured spectral range.

Using the same characterization configuration, we probe the angular response perpendicular to the mirror-symmetry plane of the layered prism structure for three different values of *θ*_*x*_ with *E*_*x*_ = 0 illumination, i.e., TE illumination at *θ*_*y*_ = 0, see Fig. 4a. Under these conditions, we observe transmission suppression near the normalized frequency *a*/*λ* ≈ 0.63 for each fixed value of *θ*_*x*_. As *θ*_*y*_ deviates from zero, the suppression weakens and disappears for ∣*θ*_*y*_∣ ≥ 5°. Accordingly, we find that the suppressed TE transmission observed in Fig. 3 persists only within a narrow angular range around the *θ*_*y*_ = 0 symmetry condition. Simulations quantitatively reproduce both the center frequency of the TE suppression at *a*/*λ* ≈ 0.63 and the angular range over which the suppression manifests. In addition, we perform transmission-mode back-focal-plane imaging to directly visualize the wavelength-dependent two-dimensional angular transmission response (see Supplementary Note 3 for details of the setup). The wavelength-dependent **k**-space measurements further confirm the symmetry defined angular selectivity of the TE transmission suppression, see Fig. 4b. At wavelengths inside the symmetry band gap, *λ* = 1340 and 1350 nm, the transmission is strongly suppressed along the *θ*_*y*_ = 0 symmetry condition and rapidly recovers away from this plane. By contrast, the suppression disappears at off-symmetry band gap wavelengths, *λ* = 1300, 1320, and 1400 nm. These back-focal-plane images provide a direct two-dimensional visualization of the symmetry band gap and confirm that the TE suppression is spectrally confined to the symmetry band gap region. Altogether, these results provide experimental evidence for the directional angular response predicted by the isofrequency analysis in Fig. 2c.

## Discussion

In conclusion, we have theoretically predicted and experimentally demonstrated a compact angle- and polarization-selective dielectric reflector whose construction is compatible with standard nanofabrication techniques and materials. By engineering a unit cell that preserves a mirror symmetry, the layered prism PhC realizes a symmetry band gap that governs the angular and polarization response along the corresponding high-symmetry orientation. While symmetry manipulation is a standard tool in the control of photonic crystal slabs and metasurfaces^54–58^, the 3D PhC’s symmetry band gap is distinguished by being a non-resonant effect, yielding robustness during fabrication that only needs to preserve the relevant symmetry rather than a finely tuned geometry. For example, despite partial etching of the bottom layer and deviations from the ideal geometry in our structure, the mirror symmetry is still preserved, which enables the symmetry band gap’s formation and the associated polarization selectivity to persist without stringent nanoscale control. While the present device employs a minimal two-period structure, the device’s response can be further engineered through systematic structural design. For example, increasing the refractive-index contrast and/or extending the number of vertical periods enhances the symmetry band gap’s width and angular selectivity, as discussed in Supplementary Note 1, providing scalable control over the operational bandwidth and angular response. Overall, the layered prism structure enables spatially selective polarization control in a single optical element that is desirable for compact photonic systems such as integrated imaging optics, optical filtering platforms, and AR display architectures. From a conceptual standpoint, our results also establish symmetry band gap engineering as a versatile framework for realizing robust, angle-resolved polarization control, yielding a promising building block for future photonic systems operating over wide spectral and angular ranges.

## Methods

### Numerical simulation

Full-wave numerical simulations were performed using both the finite-element method (FEM) and rigorous coupled-wave analysis (RCWA). For the band-structure calculations, three-dimensional periodic boundary conditions were applied to the unit cell, and the corresponding eigenfrequencies and electromagnetic Bloch field distributions were obtained. For the calculation of transmission and reflection spectra of structures with a finite height and periodicity in the *xy* plane, RCWA was employed. In the RCWA simulations, nine Fourier harmonics were included along both the *x*- and *y*-directions to ensure numerical convergence. The transmission, reflection, and absorption spectra were extracted from the scattering matrix calculated for the given incident polarization condition.

### Fabrication

1. SiN_x_film growth. Silicon nitride (SiN_*x*_) films were deposited by PECVD (Trion) at a sapphire substrate temperature of 350 °C. The film composition was controlled by adjusting the flow rate ratio of silane (SiH_4_) and nitrogen gases, measured in standard cubic centimeters per minute (sccm). By varying the SiH_4_ flow, SRN and SPN films were obtained. The optical properties of SiN_*x*_ in the visible to near-infrared spectral range were characterized by spectroscopic ellipsometry using reference films deposited on fused-silica substrates, see Supplementary Note 2. Based on the measured refractive indices, multilayer stacks were designed and fabricated by sequentially depositing alternating SRN and SPN layers on a sapphire substrate. The final structure consisted of four layers, arranged from top to bottom as SPN (500nm) / SRN (350nm) / SPN (500nm) / SRN (350nm).

2. Electron-beam lithography. Samples were solvent cleaned to reduce particles and surface residues with an acetone airbrush, rinsed with IPA, and dried with nitrogen. A dehydration bake was performed for 2 min at 170 °C. A double-coat of e-beam resist was used, starting with PMMA 495k A6 at 4k rpm baked at 170 °C for 1 min, followed by PMMA 950k A3 at 2k rpm baked at 170 °C for 1 min. Because the samples are electrically insulating, DisCharge (a spin-on anti-charging agent) was spun on at 2k rpm. The samples were written with a 1 nA beam current at a base dose of 700*μ*C cm^−2^. After writing, the DisCharge was removed with deionized water, and the PMMA was developed in a 1:3 mixture of MIBK:IPA for 70 s. The samples were rinsed with IPA, and dried with nitrogen.

3. Mask deposition & lift-off. A 150nm-thick aluminum oxide hard mask was deposited by electron-beam evaporation. To ensure clean lift-off, samples were soaked in N-methyl-2-pyrrolidone at 80 °C for over 8 h to remove residual mask material resulting from deposition-induced densification. Final cleaning was performed using acetone and IPA rinses, followed by nitrogen drying.

4. BOSCH dry etching. Due to their different stoichiometries, SRN and SPN exhibit distinct plasma-etch chemistries and etch rates. To suppress mask erosion and obtain vertical sidewalls, an etching scheme combining passivation and etching steps was used. A pre-passivation step (C_4_F_8_/Ar/O_2_) was followed by a main etching step (SF_6_/C_4_F_8_/Ar/O_2_), enabling anisotropic etching with improved sidewall control^59^.

## Supplementary information

Supplementary Information

Transparent Peer Review file

## Data Availability

The experimental and simulated data supporting the findings of this study have been deposited in Zenodo and are publicly available at https://doi.org/10.5281/zenodo.21331704^60^.
