# Supplementary material for: Polarization-dependent angularly selective dielectric reflector
Source: Nat Commun. 2026 Sep 15;17:10856. doi: 10.1038/s41467-026-77529-6 (PMC13672781; doi:10.1038/s41467-026-77529-6)
Supplement: Supplementary file 1 [file 41467_2026_77529_MOESM1_ESM.pdf]

Supplementary Information for Polarization-dependent angularly selective dielectric reflector

Hyosim Yang⋆,[1,*] Ki Young Lee⋆,[1,†] Sanghyeok Park,[1] Jiho Noh,[1] Kate Musick,[2]
Jaeyeon Yu,[1] Tomás Santiago-Cruz,[1] Chloe F. Doiron,[1,‡] and Alexander Cerjan[1,§]

[1]Center for Integrated Nanotechnologies, Sandia National Laboratories, Albuquerque, New Mexico 87185, USA
[2]Sandia National Laboratories, Albuquerque, New Mexico 87185, USA

(Dated: August 17, 2026)

Supplementary Note 1. Engineering symmetry band gap by structural design of 3D PhC

In this section, we discuss in detail the symmetry band gap of a 3D photonic crystal that considers a single mirror symmetry, and its polarization-dependent electromagnetic (EM) transmission and reflection characteristics. We show the unit-cell dispersion projected-in-k_z on the **Γ**–**X** line for the case of high refractive-index contrast between n_1 and n_2 in Supplementary Figure. 1. To present the results from a symmetry perspective, we separate the eigenvalues of the odd modes and even modes: the odd-mode eigenvalues are shown on the left as magenta dots, while the even-mode eigenvalues are shown on the right as blue dots. Note that these two sets of eigenvalues are originally overlapped in the same dispersion diagram. The symmetry band gap revealed here appears over a wider frequency range for both even and odd modes compared to the case presented in the main text.

To explore the EM scattering response associated with this unit-cell dispersion, we consider a finite stack of unit cells. Unlike the two–unit-cell stack presented in the main text, here we consider a stack of five unit cells. The TM and TE incidences are shown on the left and right, respectively. Notably, the reflectance for each polarization becomes high within the frequency domains of the symmetry band gaps where the corresponding propagation channels for coupling do not exist. In contrast, where the propagation channels for each polarization are present, the reflectance becomes low, meaning the medium becomes transparent for that polarization. On the far right of Supplementary Figure 1, we overlay the band-dispersion calculation with the EM reflectance spectra. As clearly shown in this overlaid data, within the forbidden symmetry band gaps where no propagation channels exist, the medium operates as a polarization-dependent mirror.

To demonstrate the generality of the proposed symmetry-based mechanism, we consider additional 3D PhC motifs supporting symmetry band gaps. As shown in Supplementary Figure 2**a**, the first motif consists of rectangular and square blocks with refractive indices n_1 and n_2, surrounded by air. The structural parameters are $F = 0.25$, $W = 0.3$, $h = 0.6$, $x_0 = 0.5$, $n_1 = 3.5$, and $n_2 = 1.5$. This motif preserves a single mirror symmetry M_y, producing a symmetry band gap along the corresponding **Γ**–**X** line. The calculated reflectance spectra for two- and five-period stacks show high reflectance for the $E_x = 0$ polarization channel while the symmetry-allowed $E_y = 0$ channel remains largely transmissive, consistent with the absence of symmetry-matched propagating modes in the corresponding symmetry sector.

A second motif illustrates how multiple spatial symmetries generate multiple symmetry band gaps. As shown in Supplementary Figure 2**b**, the square motif has parameters $W = 0.45$, $F = 0.35$, $n_1 = 3.5$, and $n_2 = 1.5$. Because this structure possesses M_x, M_y, and diagonal mirror symmetries, symmetry-resolved band gaps can appear along multiple mirror-invariant lines, including **Γ**–**X** and **Γ**–**M**. The corresponding five-period reflectance spectra show high reflectance within each symmetry band gap, confirming that the electromagnetic scattering response follows the symmetry-resolved band structure.

Supplementary Note 2. Optical properties of silicon nitride films

The optical properties of SRN and SPN films deposited by PECVD were characterized by spectroscopic ellipsometry (J. A. Woollam Co.'s VASE Ellipsometer). We performed measurements over the wavelength range from 400 to 1800 nm using three different angles of incidence. To improve the reliability of the extracted refractive index (n) and extinction coefficient (k), the film thicknesses were independently determined from SEM images and used as fixed parameters during the ellipsometric fitting procedure. This approach minimizes uncertainties arising from thickness–optical constant coupling and enables accurate determination of the dispersive optical properties, as shown in Supplementary Figure 3. The obtained n and k spectra were subsequently used for the design and numerical modeling of the multilayer photonic crystal structures presented in the main text.

* Email: hyang@sandia.gov; These authors contributed equally to this work
† Email: kiylee@sandia.gov; These authors contributed equally to this work
‡ Email: cfdoiro@sandia.gov
§ Email: awcerja@sandia.gov

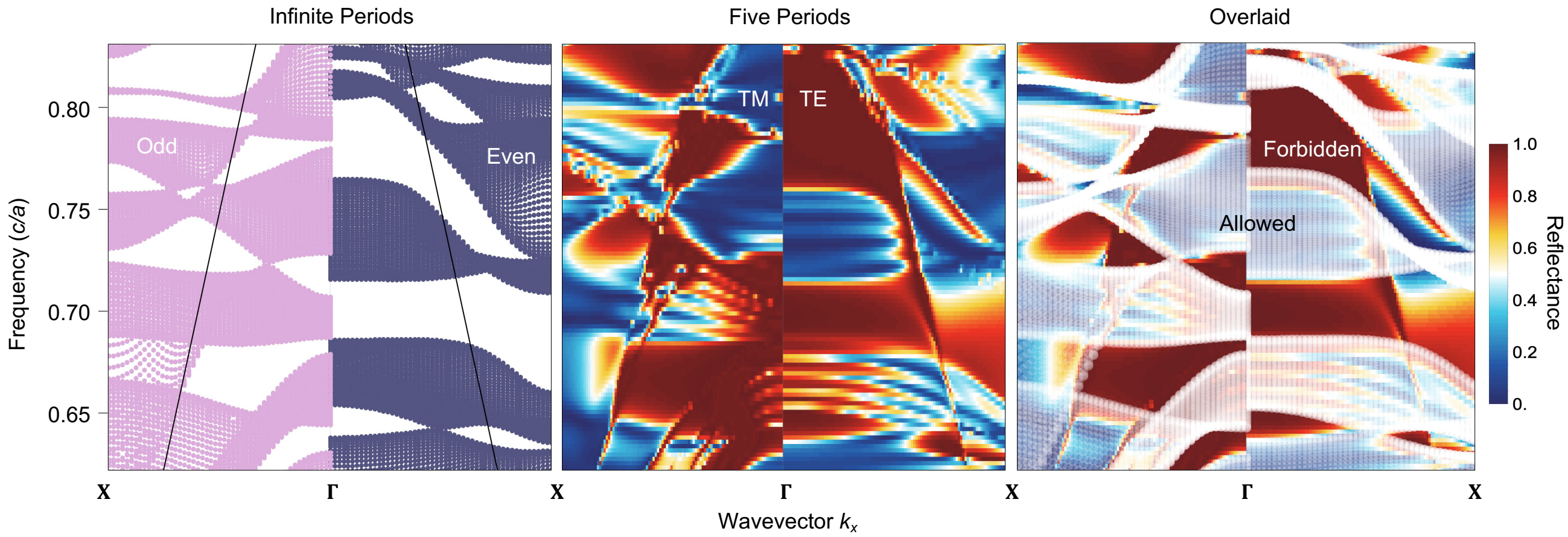

Supplementary Figure 1. **Correlation between the symmetry band gap of a 3D photonic crystal and its electromagnetic scattering response.** Left: Projected-in-k_z band structure of the cubic unit cell along the **Γ**–**X** line in the Brillouin zone. Here, the structural parameters are $n_1 = 3.5$, $n_2 = 1.5$, $h = 0.25$, and $F = 0.75$. Blue and magenta points denote modes with even and odd mirror symmetry, respectively. Although these modes physically coexist in the same domain, they are separated left and right for visualization. Center: Reflectance spectra for electromagnetic waves incident on a five-period stack of the unit cell, shown for TM (left) and TE (right) polarizations. Right: Overlay of the symmetry-resolved band dispersions with the TE/TM reflectance spectra. Regions where no dispersion points are present correspond to high reflectance, indicating the symmetry band gap.

Supplementary Note 3. Optical Measurement

We performed transmission measurements using a broadband white-light illumination setup schematically illustrated in Supplementary Figure 4**a**. A commercial fiber-coupled white-light source (Thorlabs' SLS201L) was connected to the optical system via a multimode fiber. The output beam was collimated using a lens, and an iris was placed in the collimated beam path to suppress residual diffracted and off-axis components, ensuring near plane-wave illumination along the optical axis. The polarization state of the incident light was controlled using a broadband linear polarizer positioned after the iris. The transmitted light was collected and coupled into an multimode fiber connected to a near-infrared spectrometer (Ocean Optics NIRQuest) for spectral analysis.

Angular control of the incident beam was achieved using two independent rotation stages as depicted in Supplementary Figure 4**b**. Rotation along the θ_x direction, defined with respect to the mirror-symmetry line of the triangular unit cell, was performed using an automated motorized rotation stage (Thorlabs' K10CR2) with an angular step size of 1°. Rotation along the orthogonal θ_y direction was controlled manually using a goniometer stage, also with an angular step size of 1°. A photo of the mounted 2-axis rotation stage in optical setup is the presented in Supplementary Figure 4**c**.

To probe angular selectivity perpendicular to the symmetry line, the sample was rotated by 90° within the plane of the substrate, thereby reorienting the mirror-symmetry axis of the triangular unit cell relative to the laboratory frame. With this reorientation, angular sweeps performed using the motorized rotation stage corresponded to variations along the θ_y direction in the symmetry-defined coordinate system, while the incident angle along θ_x was held fixed using the goniometer.

Prior to each measurement, the sample was carefully aligned with respect to the incident polarization and rotation axes using a CCD imaging camera to ensure that the mirror-symmetry direction of the triangular unit cell was properly oriented relative to the polarization basis. For all measurements, the transmission spectra were normalized to those obtained from an unpatterned substrate measured under identical illumination and angular conditions, thereby accounting for system response and angle-dependent coupling efficiency.

To directly map the two-dimensional angular transmission response, transmission-mode **k**-space imaging measurements were performed using a back-focal-plane imaging setup, as schematically illustrated in Supplementary Figure 5. A broadband super continuum source (NKT Photonics SuperK Compact) was used as the illumination source for the **k**-space transmission measurements. The incident polarization was controlled using a broadband linear polarizer operating in the wavelength range of 1100–1800 nm. For wavelength-selective **k**-space imaging, a hard-coated bandpass filters were inserted in the imaging path. Bandpass filters centered at 1300, 1320, 1340, 1350, and 1400 nm, each with a full width at half maximum of 12 nm, were used to acquire **k**-space transmission maps at the corresponding wavelengths (Throlabs FBH1300-12, FBH1320-12, FBH1340-12, FBH1350-12, and FBH1400-12).

The incident light was focused onto the sample using an objective lens (L1, Olympus LCPlanFL 40×, NA =

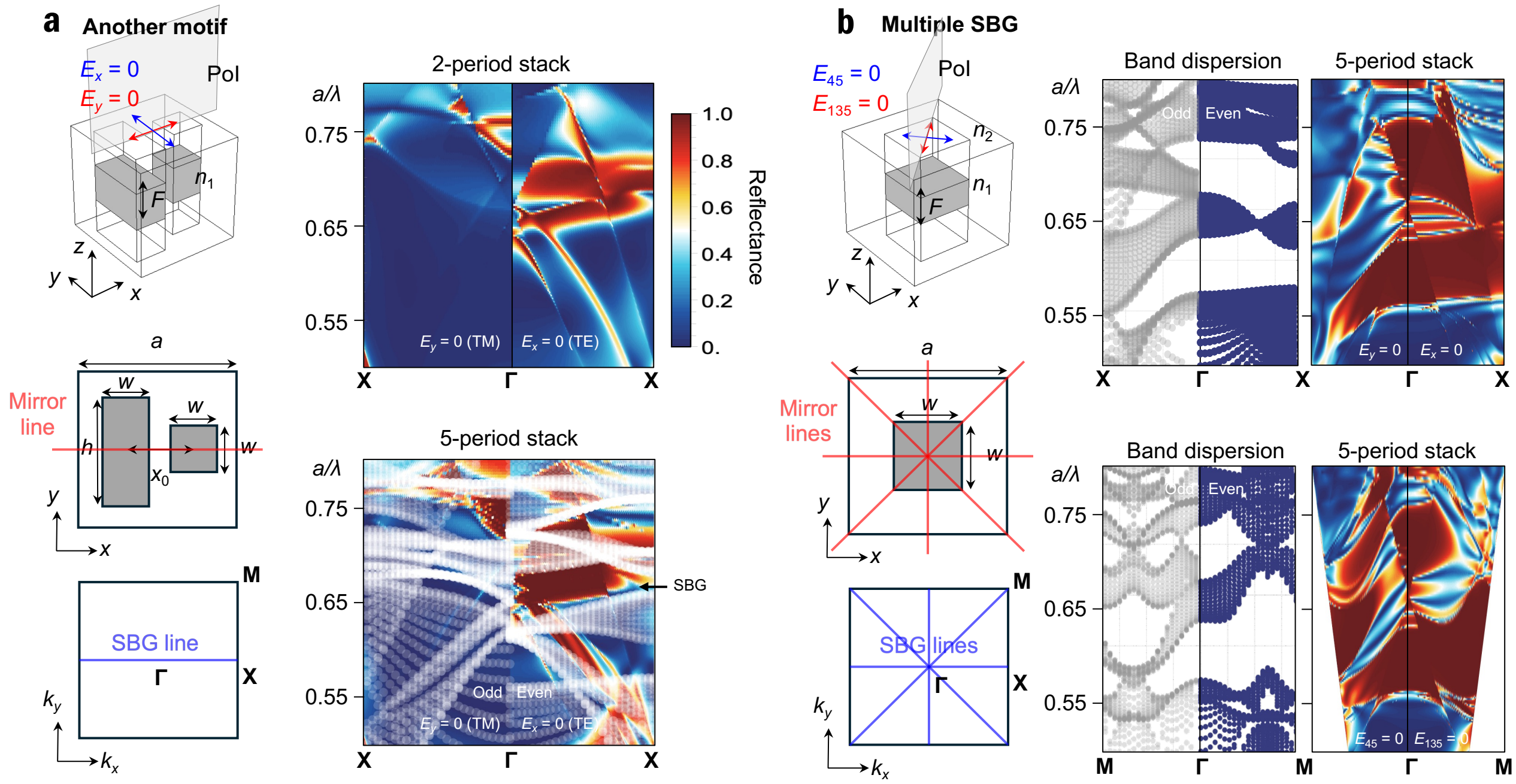

Supplementary Figure 2. **Another designs supporting symmetry band gaps in 3D photonic crystals. a**, Example of a unit cell possessing a single mirror symmetry. The left schematics show the real-space motif with the plane of incidence (PoI) for orthogonal polarization incidences and the corresponding symmetry band gap line in momentum space. The right panels show the reflectance spectra of finite stacks composed of the unit cell, including a two-period stack and a five-period stack overlaid with the symmetry-resolved projected band dispersion. The high-reflectance region appears only for the polarization channel that couples to the mirror-symmetry-forbidden sector, confirming the formation of a symmetry band gap along the corresponding mirror-invariant line. **b**, Example of multiple symmetry band gaps generated by a square motif with four mirror lines. Because the structure possesses multiple mirror symmetries in real space, symmetry band gaps are formed along the corresponding mirror-invariant lines in momentum space. The projected band dispersions and reflectance spectra are shown along both the **Γ**–**X** and **Γ**–**M** directions. In each case, the absence of symmetry-allowed bulk modes in the projected band structure correlates with high reflectance in the finite five-period stack, demonstrating that the electromagnetic scattering response directly reflects the underlying symmetry band gaps.

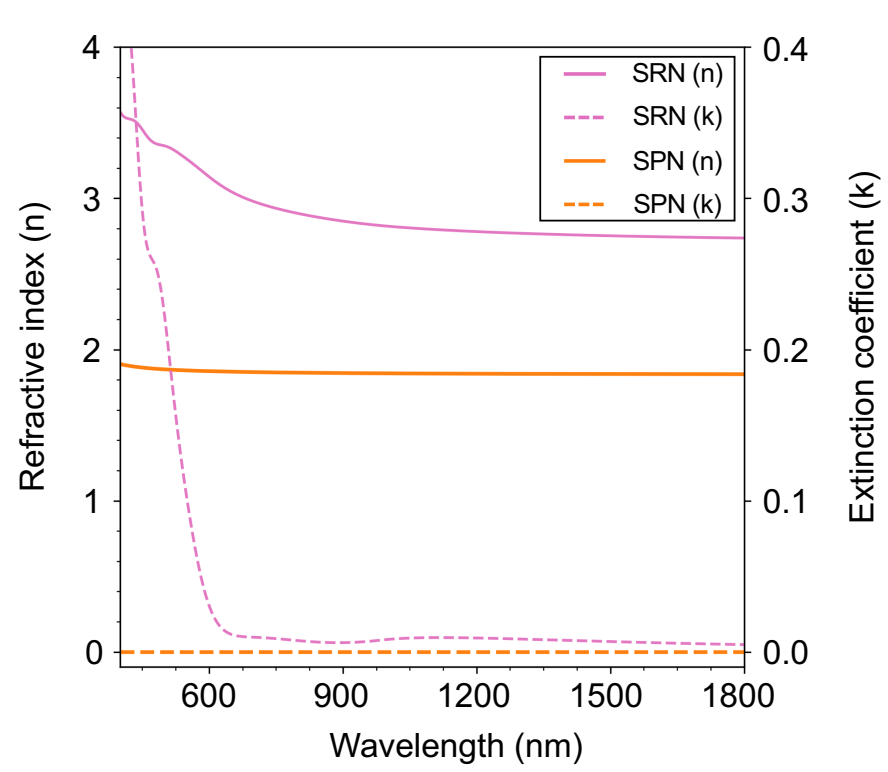

Supplementary Figure 3. **Refractive index (n) and extinction coefficient (k) of SRN and SPN films.** SRN is deposited using a gas flow ratio of $SiH_4 : N_2 = 30 : 4$, while SPN is deposited using $SiH_4 : N_2 = 2 : 4$.

0.60). The transmitted light was collected by a NIR objective lens (L2, Olympus LCPLN50× NIR, NA = 0.65) placed on the opposite side of the sample. The back focal plane of the collection objective was relayed onto the **k**-space imaging camera using a $4f$ relay system composed of two lenses with focal lengths of 200 mm (L3, Thorlabs AC254-200-C) and 150 mm (L4, Thorlabs AC254-150-C). The **k**-space images were recorded using a VSWIR camera (Alvium 1800 U-130 VSWIR). Since each position in the back-focal-plane image corresponds to a transmitted wavevector component, this configuration enables two-dimensional mapping of the transmission response along both θ_x and θ_y.

A separate real-space imaging path was used to align the patterned region of the sample with the illumination spot prior to **k**-space image acquisition. For real-space imaging, the sample was illuminated using a white-light lamp (Thorlabs SLS201L), and the real-space image was recorded using a NIR camera (CS135MUN NIR). The real-space imaging path allowed accurate positioning of the patterned region before acquiring the **k**-space transmission images.

All **k**-space transmission images were normalized by reference images obtained from an unpatterned region

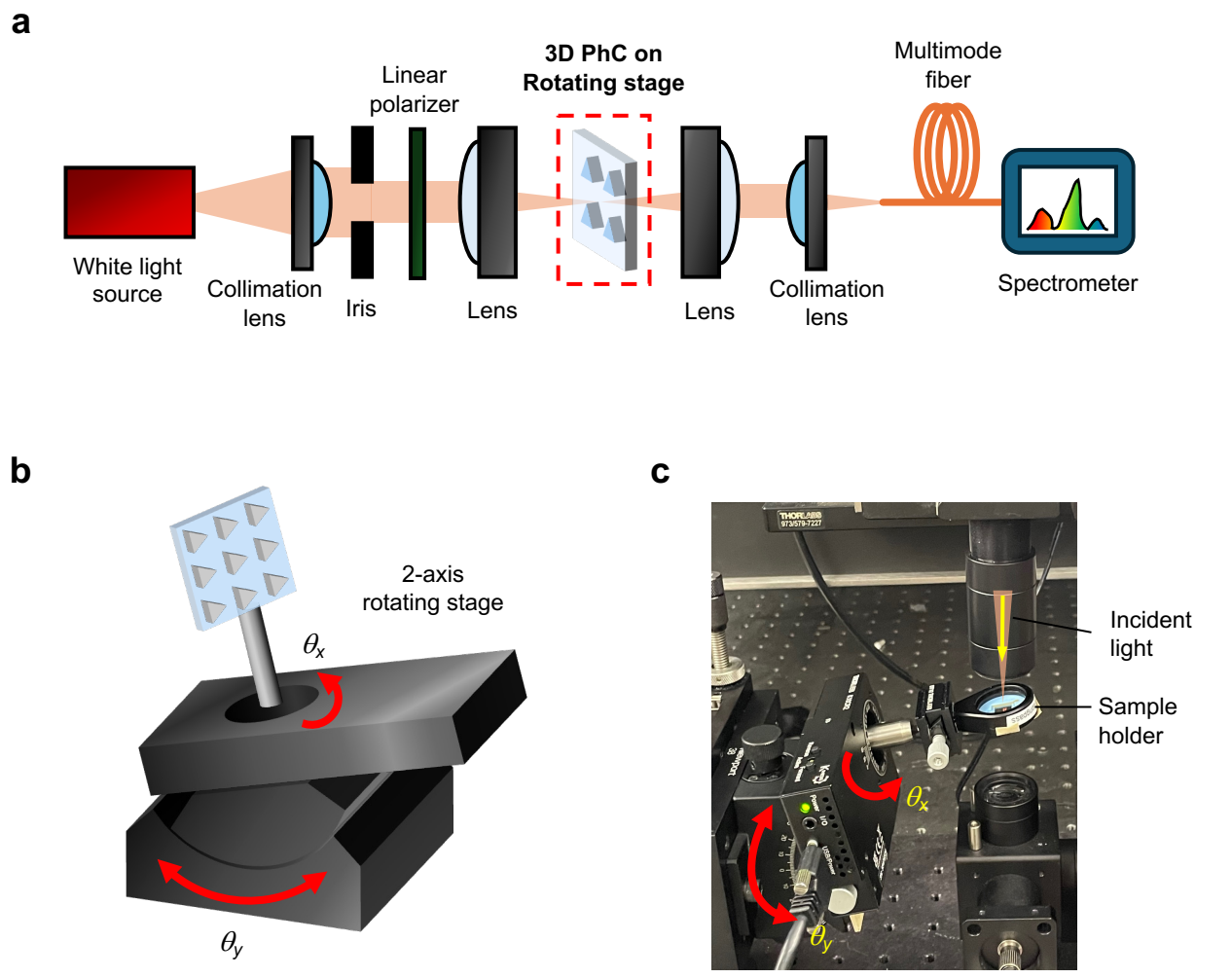

Supplementary Figure 4. **Angle resolved optical measurement system.** **a**, Schematic of the optical measurement system. **b**, Schematic of the 2-axis rotating stage. **c**, A photo of the mounted rotating stages in the measurement system.

of the same substrate under identical illumination, polarization, wavelength-filtering, and imaging conditions. This normalization accounts for the system response, substrate transmission, and angle-dependent collection efficiency.

We further measured the transmission-mode back-focal-plane response for the orthogonal incident polarization, $E_y = 0$, as shown in Supplementary Figure 6. For $E_y = 0$ incidence in $\theta_x = 0$ plane, this corresponds to TM polarization, whereas the main text focuses on the $E_x = 0$ (TE at $\theta_y = 0$) incidence because the symmetry-band-gap (SBG) effect appears in the TE channel. The $E_y = 0$ notation is used here because it remains a rigorous polarization definition for arbitrary oblique incidence. Experimental and simulated isofrequency substrate-normalized transmission images are shown in the top and bottom rows, respectively, for five representative wavelengths, $\lambda = 1300$, 1320, 1340, 1350, and 1400 nm.

Near the SBG spectral range, particularly around $\lambda =$ 1340–1350 nm, the $\theta_y = 0$ line remains largely transmissive for $E_y = 0$ incidence. This behavior contrasts with the suppressed transmission observed for the orthogonal $E_x = 0$ incident polarization in the main text, confirming that the angular transmission suppression is polarization selective.

We note that the agreement between experiment and simulation is less direct for the $E_y = 0$ data than for the $E_x = 0$ data shown in the main text. This discrepancy appears to originate from a blue shift of the TM-like transmission feature in the experiment. As shown in Fig. 3 of the main text, the simulated TM feature lies on the longer-wavelength side near the SBG spectral region, whereas the corresponding experimental feature appears closer to the SBG region near $\lambda = 1350$ nm. Although the origin of this shift is not fully understood, the same trend explains the back-focal-plane data in Supplementary Figure 6: the dark transmission feature along the $\theta_x = 0$ line, which appears near $\lambda = 1400$ nm in the simulation, is observed experimentally at the blue-shifted wavelengths around $\lambda = 1340$–1350 nm.

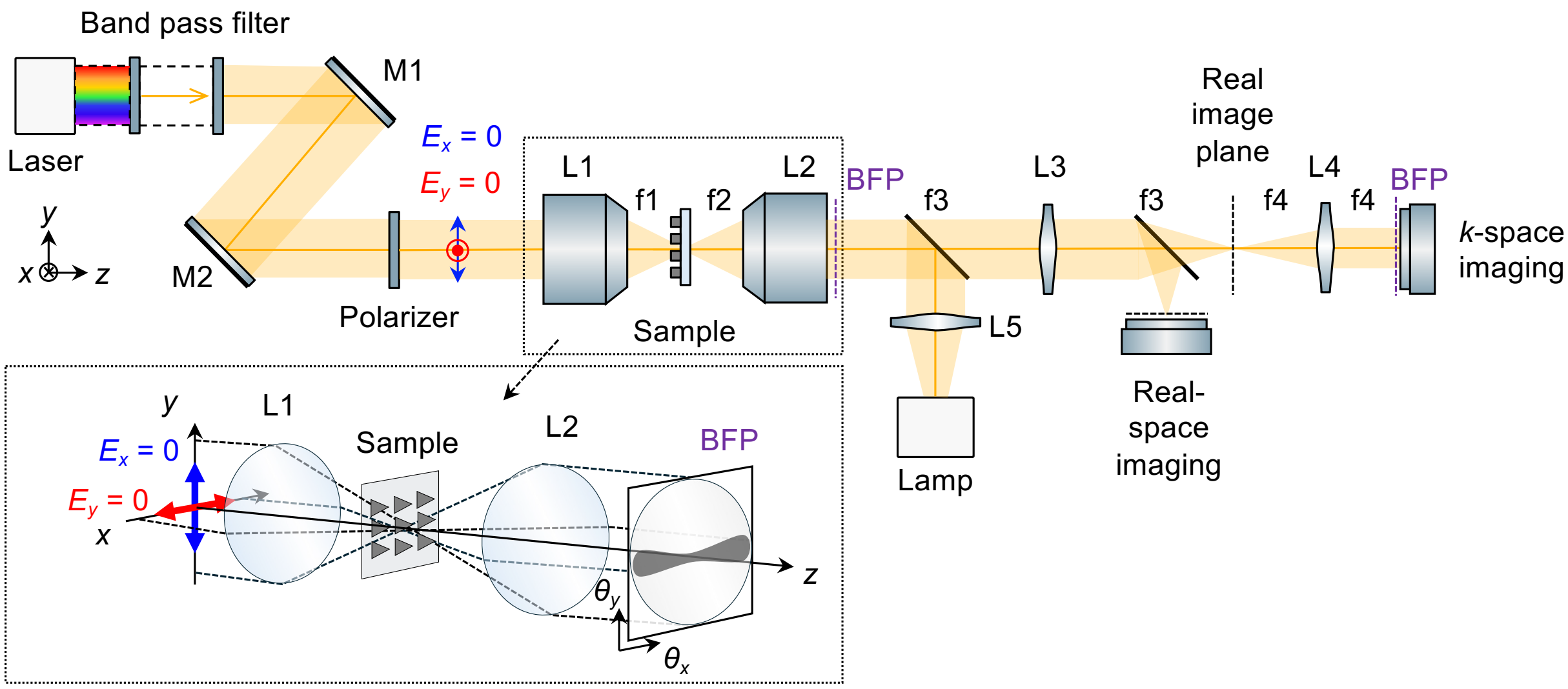

Supplementary Figure 5. **Transmission-mode back-focal-plane imaging setup based on a $4f$ relay system.** Schematic of the back-focal-plane (BFP) imaging setup. The beam path was folded by two mirrors (M1 and M2) while maintaining a parallel optical axis through the system. The incident polarization was controlled as TE or TM using a linear polarizer. The incident light was focused onto the sample by the objective lens L1, and the transmitted light was collected by the objective lens L2. The BFP of L2 was relayed onto the **k**-space imaging camera using relay lenses L3 and L4. The enlarged view highlights the imaging relation around the sample, where the real-space sample plane and the Fourier-space BFP plane are shown on opposite sides of the objective pair. At the BFP of L2, the transmitted angular distribution is encoded as a **k**-space intensity pattern, with positions in the BFP corresponding to the angular coordinates θ_x and θ_y in momentum space. A separate real-space imaging path was implemented using a white-light lamp and a lens (L5, $f = 75$ mm) to provide Köhler illumination for sample alignment.

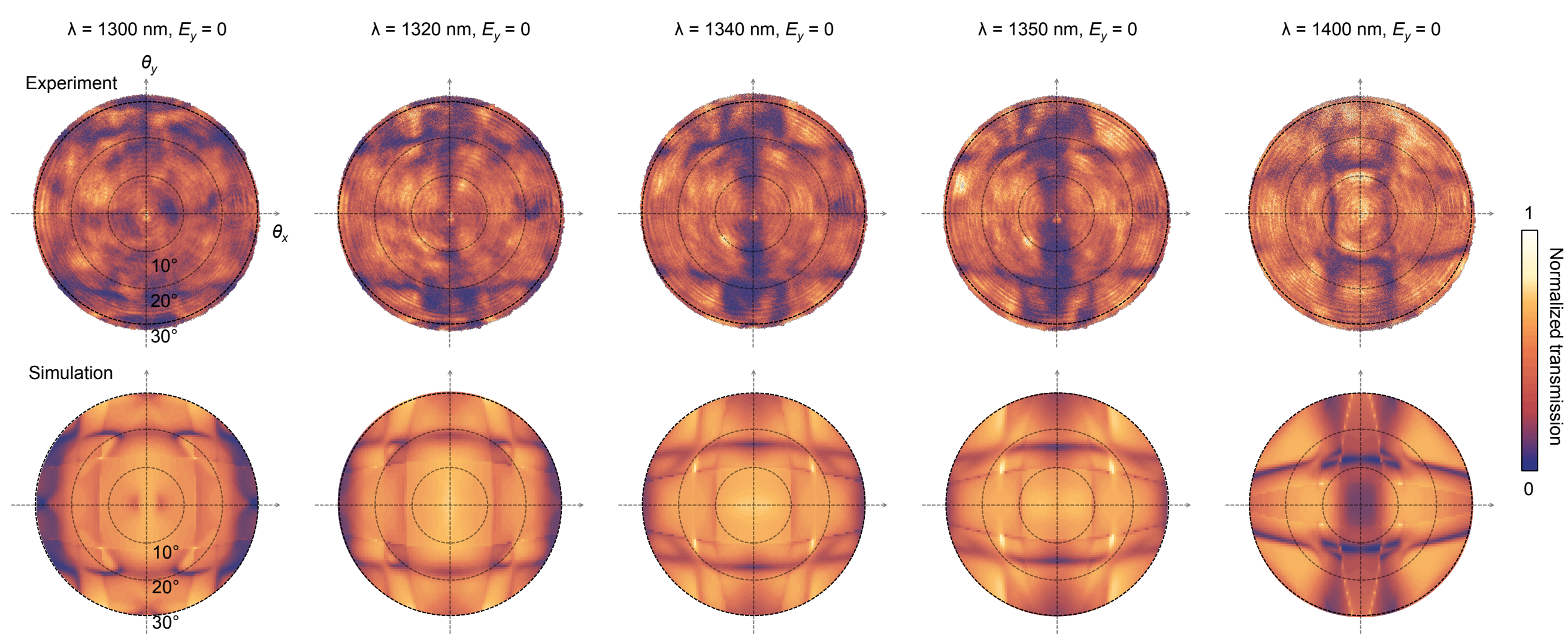

Supplementary Figure 6. **Normalized transmission images of back-focal-plane for the $E_y = 0$ incident polarization.** Experimental (top row) and simulated (bottom row) isofrequency substrate-normalized transmission images for $E_y = 0$ polarization at wavelengths from $\lambda = 1300$ to 1400 nm.
