# Supplementary material for: Polarization-dependent angularly selective dielectric reflector
Source: Nat Commun. 2026 Sep 15;17:10856. doi: 10.1038/s41467-026-77529-6 (PMC13672781; doi:10.1038/s41467-026-77529-6)
Supplement: Supplementary file 2 [file 41467_2026_77529_MOESM2_ESM.pdf]

nature portfolio

Peer Review File

Polarization-dependent angularly selective dielectric reflector

Corresponding Author: Dr Alexander Cerjan

This file contains all reviewer reports in order by version, followed by all author rebuttals in order by version.

Attachments originally included by the reviewers as part of their assessment can be found at the end of this file.

Version 0:

Reviewer comments:

Reviewer #1

(Remarks to the Author)
Please see the uploaded file.

Reviewer #2

(Remarks to the Author)
The authors explore a truncated 3D photonic crystal with a symmetry band gap over a large range of incident angles in a chosen horizontal direction. The result is a compact optical thin film with peculiar and surprising combination of responses with respect to polarization, angle, and frequency. The engineer is based primarily in symmetry-based concepts, while not relying on resonance, making it potentially powerful and reliable. The concept (related to Ref. [44]) is still relatively fresh and unexplored in the literature, and this work, in my opinion, shows there may be more to this concept and its implementation than previously appreciated. This work is quite compelling in terms of simplicity and flexibility of implementation, compared to past results and alternative approaches. I find it reasonably likely to spark renewed interest in these ideas. The writing and presentation are excellent. I am happy to recommend publication of this article. I have only a few minor comments below.

Minor comments:
1. There seems to be mixture of labeling of incident conditions—sometimes “TE/TM”, sometimes “Exz”, sometimes “Ex=0”, etc. A more consistent labeling would be helpful.
2. The following passage has confusing wording to me: “In wavevector space, this mirror symmetry defines a high-symmetry plane at ky=0, along which the PhC’s Bloch eigenmodes can be classified as either even or odd with respect to My.” I believe I understand—for modes along ky=0, there is a REAL-space symmetry captured by My. I agree with this. I am confused by the language “In wavevector space” at the beginning—this symmetry exists in real space for specific incident angles.
3. While I find Fig S1 quite valuable, I find myself wanting a bit more clarity on the requirements vs freedoms with respect to symmetry. If I understand correctly, the authors want to claim the following: The reflection symmetry My is required for the symmetry bandgap, but no other symmetries are required (they are free to keep or break). But, unless I missed it, I did not see explicit clarification of this point. Am I correct in assuming it is just convenient / gives more design flexibility to do break mirror symmetry in the x direction, for eample? If so, is it ‘practical requirement’ to have such flexibility to find a design? Part of what I am getting at is to explicitly clarify the role of symmetries and design paradigm, and part of what I am asking is about how generic / easy it is to find such behavior. Can the authors briefly comment/clarify?
4. The agreement between simulation and experiment is quite good, in my view, for the high symmetry plane of interest (figure 3). While still quite reasonable, it appears notably less good along the theta_y direction (figure 4), especially some odd disconnects right at theta_y = 0 compared to the theta_x plane. This is minor, and does not impact the evidence / claims of the main paper. However, some explanation/discussion as to this discrepancy in agreement may be appropriate.
5. I agree with the point in the conclusion of the potential practical advantages of this being a non-resonant effect. Some mention earlier may also be appropriate, as it is a distinguishing aspect of this work and the idea of a symmetry bandgap. However, I would also be curious if such an effect (over some resonant bandwidth) is expected to be feasible with resonant devices? I notice the authors have this related expertise, and hence may comment. For instance, the “flat band” device in Ref. [R1] seems to have the potential quite comparable in behavior, but with a distinct mechanism (and much narrower bandwidth). Or, is there some fundamental barrier to achieving something like this with resonant features with low Q-factor to make it more comparable to the 20nm bandwidth observed here? Some more comparison/discussion, and especially and

explicit mention of whether the equivalent is expected to be achievable with a resonant mechanisms (albeit less practically) would be helpful.
[R1] H.S. Nguyen, et al. “Symmetry Breaking in Photonic Crystals: On-Demand Dispersion from Flatband to Dirac Cones”, Physical Review Letters 120, 066102, (2018).

Reviewer #3

(Remarks to the Author)
The manuscript proposes a truncated three-dimensional photonic crystal that exhibits a symmetry band gap along a high-symmetry direction, and experimentally demonstrates its operation as a compact, all-dielectric optical element capable of simultaneous polarization separation and angular selectivity. The experimental results are generally consistent with the theoretical design. In particular, polarization-dependent angular selectivity has, in my view, already been demonstrated in previous works, including Science 343, 1499 (2014), ACS Photonics 5, 4125 (2018), and Nature Photonics 7, 791 (2013). More broadly, it is well known that anisotropic structures, or structures under oblique illumination, can exhibit polarization-dependent spectral responses. Therefore, the statement in the abstract that this work “extends polarization control into the angular domain” appears somewhat misleading or at least overstated. On the other hand, although the polarization-dependent angular spectrum here may differ from prior works, I can not find the unique value in terms of applications and I am concerned about its broad interest in the society. Considering the high-quality of Nature Communications, I can not recommend for the publication.

Other comments are following:

(1)The authors are supposed to discuss more thoroughly recent progresses on simultaneous polarization separation and angular selectivity in the introduction. In particular, the authors should provide a more detailed comparison with prior studies for claiming their novelty in design or applications.
(2)The authors claim that a symmetry band gap will lead to high reflection and a disappeared band gap will allow the transmission. However, even in the small-angle region where a cluster of odd modes exists and the infinite crystal no longer appears to have a clear band gap, the two-period structure still show high reflection. The relationship between high reflection and the presence or absence of the symmetry band gap therefore needs to be explained more carefully.
(3)In experiment, the most common polarization bases are linear polarization, such as s/p or x/y polarization, and circular polarization. By contrast, the polarization condition defined by $E_x=0$ when θ_x and θ_y are nonzero, as used in fig. 2b, appears rather specific and may be of limited practical relevance.
Although Fig. 4 shows an angle-dependent spectrum, I am not fully convinced that it demonstrates practically useful angular selectivity, because other resonant features are also present in the wavelength range of interest and may introduce crosstalk. What’s more, to better show the angular selectivity, the authors are supposed to present a series of two-dimension reflection in momentum space under different incident wavelengths.

Version 1:

Reviewer comments:

Reviewer #1

(Remarks to the Author)
The authors have revised the manuscript carefully. Now, I recommend the acceptance.

Reviewer #2

(Remarks to the Author)
The authors have answer all of my concerns very well. I am happy to recommend publication.

Reviewer #3

(Remarks to the Author)
The authors have substantially addressed my previous concerns. In particular, they have clarified the novelty and physical mechanism of the proposed device by extensive supplementary results and detailed comparisons with prior works. I appreciate the efforts they made and recommend publication of the revised manuscript in this form.

Open Access This Peer Review File is licensed under a Creative Commons Attribution 4.0 International License, which permits use, sharing, adaptation, distribution and reproduction in any medium or format, as long as you give appropriate credit to the original author(s) and the source, provide a link to the Creative Commons license, and indicate if changes were made.
In cases where reviewers are anonymous, credit should be given to 'Anonymous Referee' and the source.
The images or other third party material in this Peer Review File are included in the article's Creative Commons license, unless indicated otherwise in a credit line to the material. If material is not included in the article's Creative Commons license and your intended use is not permitted by statutory regulation or exceeds the permitted use, you will need to obtain permission directly from the copyright holder.
To view a copy of this license, visit https://creativecommons.org/licenses/by/4.0/

Author Response Letter to the Reviewer Comments

Manuscript: NCOMMS-26-021464-T
Title: Polarization-dependent angularly selective dielectric reflector
Authors: Hyosim Yang, Ki Young Lee, Sanghyeok Park, Jiho Noh, Kate Musick, Jaeyeon Yu, Tomás Santiago-Cruz, Chloe F. Doiron, Alexander Cerjan

Dear Editor and Reviewers,

We are grateful to the editor and the reviewers for their thoughtful and constructive feedback. Their careful comments helped us improve the manuscript in several important aspects, including the physical explanation, comparison with prior work, consistency of notation, and additional numerical and experimental validation. We have carefully revised the manuscript accordingly, as detailed below.

In the following, the reviewer comments are shown in **Black**, and our responses are provided in **Blue**; Author Response (AR). All corresponding changes in the manuscript are **highlighted in yellow**, with positions indicated based on the revised version.

Reviewer A

In the submitted manuscript, the authors theoretically and experimentally realized polarization-dependent angular selection in 3D photonic crystals (PhCs). When incident light is aligned along the high-symmetry direction, the symmetry photonic band gap (PBG) produces polarization-dependent reflection and transmission. By contrast, deviation from this orientation lifts the symmetry constraint, resulting in the disappearance of polarization selectivity. Overall, I think this manuscript is presented in a good shape in terms of sound theoretical foundations, rich visualizations, and rigorous validations. Nonetheless, the writing quality of the manuscript could be further improved. After the authors revise the manuscript based on the following comments, I would recommend the acceptance of this manuscript for publication in Nature Communications.

(AR-A0) We thank the reviewer for recommending the acceptance of our manuscript and for providing constructive comments to improve its writing and presentation. We have thoroughly revised the manuscript in response to each of the reviewer's comments, as detailed below.

1) From the results in Fig. 2(a), the PBG for odd channel is distinct from that from even channel. Please provide the underlying physical reason.

(AR-A1) We appreciate this important comment, as it addresses the physical origin of the symmetry-resolved band gaps. The full 3D PhC considered here does not possess a complete photonic band gap (PBG). However, along the $k_y = 0$ high-symmetry line, the mirror symmetry M_y allows the Bloch modes to be separated into even and odd symmetry families.

These two symmetry families generally have different field profiles and nodal structures, and therefore form distinct modal dispersions, similar to different symmetry families in waveguides or photonic-crystal slabs. As a result, a band gap along $k_y = 0$ can appear within one symmetry sector even though the full 3D PhC remains gapless. This is what we call the symmetry band gap.

This symmetry-resolved band gap then determines the polarization-dependent scattering response: when the incident polarization has no symmetry-matched propagating channel inside the PhC, transmission is suppressed.

Revisions made:

- We revised the discussion around Fig. 2(a) to clarify why the even and odd symmetry channels form distinct band structures and symmetry band gaps.

(Main Text: Line 124 on page 2)
Although the full 3D PhC remains gapless, the even- and odd-in-E_y modes with $k_y = 0$ belong to distinct M_y symmetry sectors with different field parity and nodal structures, so each symmetry sector can exhibit its own band gaps in certain frequency ranges.

2) Without loss of generality, I suggest the authors utilize pillars with alternative geometries (e.g., T-shaped pillars) in the SM to realize polarization-dependent angular selection.

(AR-A2) We appreciate this suggestion, especially because it emphasizes the broad design possibilities of our approach. The triangular pillar geometry is not essential to our mechanism; the key requirement is the mirror symmetry that enables even/odd mode classification and a symmetry band gap.

We have added an alternative mirror-symmetric geometry in the revised Supplemental Information, confirming that polarization-dependent angular selection can be realized beyond the triangular pillar design.

Revisions made:

- We added a Supplementary figure and discussion, along with a brief main-text statement on this design generality.

(New supplemental figure S2 for showing additional motif designs)

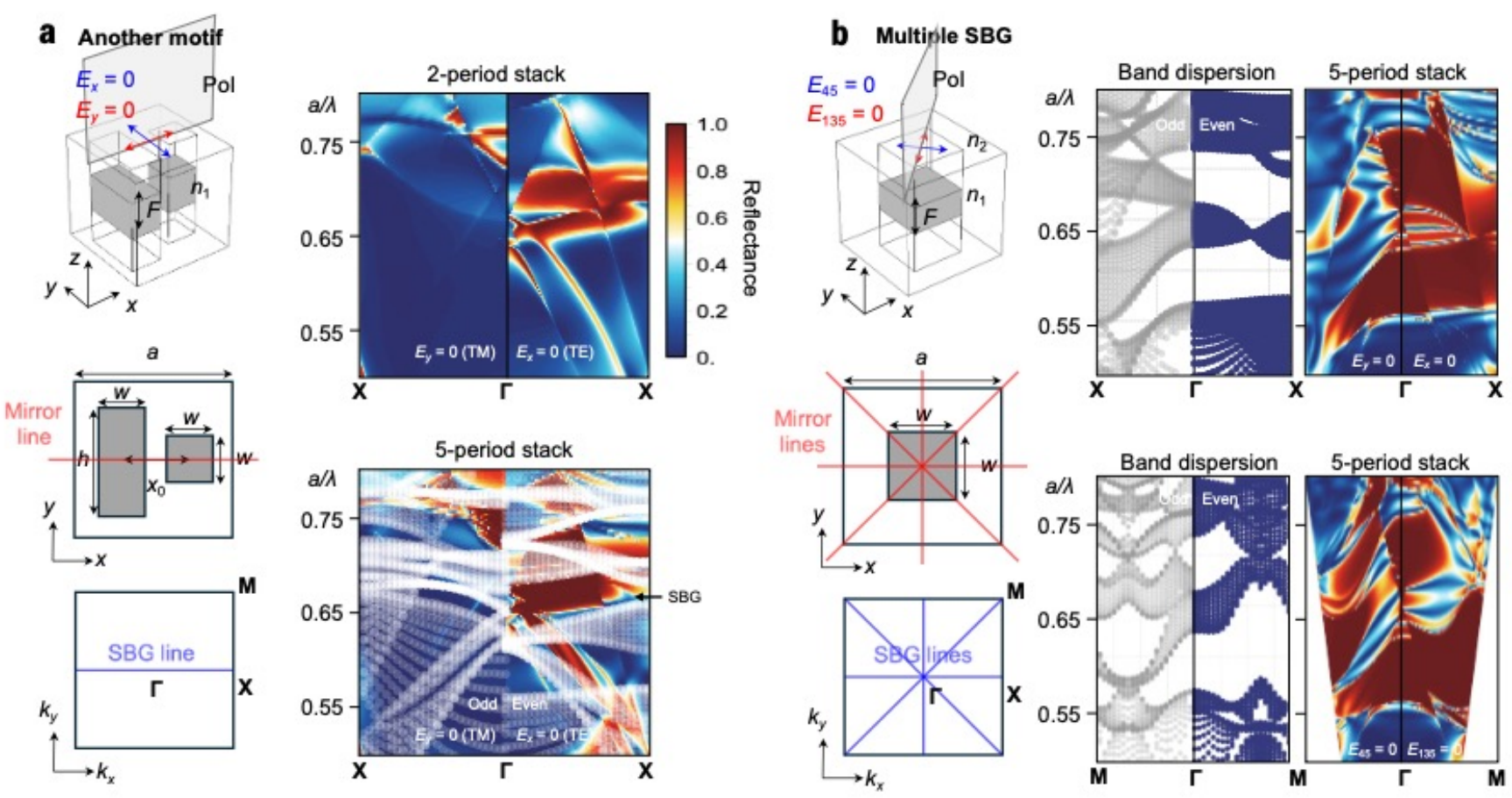

FIG. S2. **Another designs supporting symmetry band gaps in 3D photonic crystals.** **a**, Example of a unit cell possessing a single mirror symmetry. The left schematics show the real-space motif with the plane of incidence (PoI) for orthogonal polarization incidences and the corresponding symmetry band gap line in momentum space. The right panels show the reflectance spectra of finite stacks composed of the unit cell, including a two-period stack and a five-period stack overlaid with the symmetry-resolved projected band dispersion. The high-reflectance region appears only for the polarization channel that couples to the mirror-symmetry-forbidden sector, confirming the formation of a symmetry band gap along the corresponding mirror-invariant line. **b**, Example of multiple symmetry band gaps generated by a square motif with four mirror lines. Because the structure possesses multiple mirror symmetries in real space, symmetry band gaps are formed along the corresponding mirror-invariant lines in momentum space. The projected band dispersions and reflectance spectra are shown along both the **Γ**–**X** and **Γ**–**M** directions. In each case, the absence of symmetry-allowed bulk modes in the projected band structure correlates with high reflectance in the finite five-period stack, demonstrating that the electromagnetic scattering response directly reflects the underlying symmetry band gaps.

(Supplementary Information: Line 43 on page 1)
To demonstrate the generality of the proposed symmetry-based mechanism, we consider additional 3D PhC motifs supporting symmetry band gaps. As shown in Fig. S2**a**, the first motif consists of rectangular and square blocks with refractive indices n_1 and n_2, surrounded by air. The structural parameters are $F = 0.25$, $W = 0.3$, $h = 0.6$, $x_0 = 0.5$, $n_1 = 3.5$, and $n_2 = 1.5$. This motif preserves a single mirror symmetry M_y, producing a symmetry band gap along the corresponding **Γ**–**X** line. The calculated reflectance spectra for two- and five-period stacks show high reflectance for the $E_x = 0$ polarization channel while the symmetry-allowed

$E_y = 0$ channel remains largely transmissive, consistent with the absence of symmetry-matched propagating modes in the corresponding symmetry sector.

(Main Text: Line 211 on page 4)
A more detailed explanation of the causal relation between a symmetry band gap and polarization-dependent electromagnetic scattering is provided in Supplementary Information Sec. SI, along with examples of additional structures that achieve this effect.

3) In Fig. 2(a), the authors give the results for two periods. To better understand the transition from finite-period to infinite-period cases, the results for three and five periods should be given in the SM.

(AR-A3) We appreciate this suggestion. This point is closely related to the design-generality discussion in AR-A2, and we have addressed both issues together in the revised Supplementary Information. Specifically, we added Figs. S1 and S2, together with the corresponding discussion in Supplementary Information Sec. SI, to show the finite-period cases of the reflectance spectra and their connection to the symmetry-resolved band structures.

Revisions made:

- We added Figs. S1 and S2 and the corresponding discussion in Supplementary Information Sec. SI. Please also refer AR-A2.

4) On page 2, the authors mention that "when the materials' index contrast is sufficiently large, $\Delta n \geq 0.8$". The authors need to clarify whether n_1 or n_2 is fixed.

(AR-A4) We thank the reviewer for pointing this out. In the analysis referred to here, the higher refractive index n_1 was fixed while the lower refractive index n_2 was varied.

Revisions made:

- We have also revised the corresponding sentence to clarify this point in the main text.

(Main Text: Line 122 on page 2)
when the materials' index contrast is sufficiently large, $\Delta n \gtrsim 0.8$ for a fixed higher-index material with $n_{high} = 3.5$.

5) Over the past two decades, researchers achieved angle-independent PBGs [Appl. Phys. Lett. 83, 5386 (2003); Appl. Phys. Lett. 112, 041902 (2018)] and redshift PBGs [Phys. Rev. Appl. 10, 064022 (2018)] in 1D PhCs containing metamaterials. As the incident angle increases, the redshift PBGs shifts towards longer wavelengths. The authors could consider involving these works in the introduction section.

(AR-A5) We thank the reviewer for bringing these relevant studies to our attention. We agree that prior works on angle-independent and engineered angle-dependent photonic band gaps in metamaterial-based one-dimensional photonic crystals are highly relevant to the wide-field-of-view aspect of polarization-dependent optical devices.

Revisions made:

- Accordingly, we have added these references to the Introduction, together with representative wide-field-of-view metasurface studies.

(Main Text: Line 45 on page 1)
While prior metasurface, metamaterial, and photonic crystal–based approaches have demonstrated individual aspects of these capabilities, including wide-field polarization control, engineered angular responses, and compact analog image processing [38–52],...

(Added references are listed below:

[38] H. Jiang, H. Chen, H. Li, Y. Zhang, and S. Zhu, Omni-directional gap and defect mode of one-dimensional photonic crystals containing negative-index materials, Applied Physics Letters 83, 5386 (2003)
[44] F. Wu, G. Lu, C. Xue, H. Jiang, Z. Guo, M. Zheng,C. Chen, G. Du, and H. Chen, Experimental demonstration of angle-independent gaps in one-dimensional photonic crystals containing layered hyperbolic metamaterials and dielectrics at visible wavelengths, Applied Physics Letters 112, 041902 (2018)
[45] F. Wu, G. Lu, Z. Guo, H. Jiang, C. Xue, M. Zheng, C. Chen, G. Du, and H. Chen, Redshift gaps in one-dimensional photonic crystals containing hyperbolic metamaterials, Phys. Rev. Appl. 10, 064022 (2018))

6) Other small issues:
- In Fig. 2(a), the values of 0.25 and -0.25 do not correspond to the midpoint between points X and Γ.
- In Figs. 2(b) and 2(c), please add the unit of the incident angle.

(AR-A6) We thank the reviewer for catching these errors. We have corrected the tick labels in Fig. 2(a) and added the angle units in Figs. 2(b,c).

Reviewer B

The authors explore a truncated 3D photonic crystal with a symmetry band gap over a large range of incident angles in a chosen horizontal direction. The result is a compact optical thin film with peculiar and surprising combination of responses with respect to polarization, angle, and frequency. The engineer is based primarily in symmetry-based concepts, while not relying on resonance, making it potentially powerful and reliable. The concept (related to Ref. [44]) is still relatively fresh and unexplored in the literature, and this work, in my opinion, shows there may be more to this concept and its implementation than previously appreciated. This work is quite compelling in terms of simplicity and flexibility of implementation, compared to past results and alternative approaches. I find it reasonably likely to spark renewed interest in these ideas. The writing and presentation are excellent. I am happy to recommend publication of this article. I have only a few minor comments below.

(AR-B0) We thank the reviewer for recognizing the novelty and potential impact of our symmetry-based, non-resonant approach, and for providing thoroughtful and constructive comments. These comments gave us a valuable opportunity to further clarify the central concept and improve the presentation of the manuscript. We have addressed the reviewer's comments individually, as detailed below.

Minor comments:

1. There seems to be mixture of labeling of incident conditions—sometimes "TE/TM", sometimes "Exz", sometimes "Ex=0", etc. A more consistent labeling would be helpful.

(AR-B1) We thank the reviewer for raising this important point. We agree that since our manuscript describes the interaction between symmetry-classified PhC modes and externally incident polarization states, a consistent definition of the incident polarization basis is essential for avoiding confusion.

Revisions made:

- We revised the beginning of the Results section to define the incident polarization notation more thoroughly, and explicitly make the connection to both TE/TM and s/p definitions of polarization.
- Following this discussion, the manuscript adopts a single convention, with edits to the text and figures to ensure consistency.

(Main Text: Line 141 on page 3)
For frequencies within the symmetry band gap, only odd-in-E_y modes are available along the $\mathbf{\Gamma}$–$\mathbf{X}$ high-symmetry line. Therefore, any even-in-E_y incident light has a vanishing symmetry overlap with these odd-in-E_y modes and cannot couple to a propagating mode inside the PhC, resulting in suppressed transmission. For incoming light with a wavevector in the xz-plane, the polarization condition $E_x = 0$ corresponds to an incident field with only an even-in-E_y electric-field component, independent of θ_x (see Fig. 1**a**), as the field profile of the incoming plane wave has no dependence on y. Note that for $k_y = 0$, the $E_x = 0$ polarization is the transverse-electric (TE), or s-polarized, free-space channel. Thus, TE incident light is expected to be suppressed within the symmetry band gap. In contrast, transverse-magnetic (TM), or p-polarized, incident light with $\theta_y = 0$ satisfies $E_y = 0$, which corresponds to non-zero E_{xz} and is not symmetry-forbidden from coupling to the available odd-in-E_y modes, allowing transmission through the PhC. When the incident light is not aligned with the high-symmetry line, $\theta_y \neq 0$, the mirror symmetry constraint is lifted, and both incident polarizations can couple to the modes of the 3D PhC. In the following, we use the free-space polarization basis $E_x = 0$ and $E_y = 0$ to denote the two incident polarization channels for arbitrary incident angles (θ_x, θ_y), which maintains a straightforward connection to the separable symmetry sectors of the layered prism 3D PhC when $\theta_y = 0$.

2. The following passage has confusing wording to me: "In wavevector space, this mirror symmetry defines a high-symmetry plane at ky=0, along which the PhC's Bloch eigenmodes can be classified as either even or odd with respect to My." I believe I understand—for modes along ky=0, there is a REAL-space symmetry captured by My. I agree with this. I am confused by the language "In wavevector space" at the

beginning—this symmetry exists in real space for specific incident angles.

(AR-B2) We thank the reviewer for this precise clarification. We agree that M_y is a real-space mirror symmetry of the crystal structure, and that the relevant statement is that this real-space symmetry defines invariant subspaces in wavevector space where Bloch eigenmodes can be classified by their M_y parity. More generally, a real-space point-group operation S maps wavevectors as $\mathbf{k} \to (S^{-1})^{\top}\mathbf{k}$, up to a reciprocal lattice vector. For a mirror operation, $(S^{-1})^{\top} = S$, so the real-space symmetry $M_y(x, y, z) = (x, -y, z)$ corresponds to the wavevector-space mapping $M_y(k_x, k_y, k_z) = (k_x, -k_y, k_z)$. The plane $k_y = 0$ is therefore invariant under this operation, allowing Bloch modes on this plane to be classified as even or odd with respect to the real-space mirror symmetry M_y. We have revised the text to make this connection explicit.

Revisions made:

- We revised the corresponding sentence in the main text to clarify that although M_y is initially identified as a real-space symmetry, it also defines a wavevector-space symmetry, which in turn defines the high-symmetry plane at $k_y = 0$ that is crucial to our design.

(Main Text: Line 109 on page 2)
In wavevector space, this mirror symmetry acts as $M_y(k_x, k_y, k_z) = (k_x, -k_y, k_z)$, so the plane $k_y = 0$ is invariant under M_y and defines a high-symmetry plane. As such, for $k_y = 0$, the PhC's Bloch eigenmodes can be classified through their spatial profiles as either even or odd with respect to the real-space mirror symmetry M_y.

3. While I find Fig S1 quite valuable, I find myself wanting a bit more clarity on the requirements vs freedoms with respect to symmetry. If I understand correctly, the authors want to claim the following: The reflection symmetry My is required for the symmetry bandgap, but no other symmetries are required (they are free to keep or break). But, unless I missed it, I did not see explicit clarification of this point. Am I correct in assuming it is just convenient / gives more design flexibility to do break mirror symmetry in the x direction, for example? If so, is it 'practical requirement' to have such flexibility to find a design? Part of what I am getting at is to explicitly clarify the role of symmetries and design paradigm, and part of what I am asking is about how generic / easy it is to find such behavior. Can the authors briefly comment/clarify?

(AR-B3) We appreciate this important comment. Yes, the reflection symmetry M_y is required for a symmetry band gap to form along $k_y = 0$, but no other symmetries are required. And no, it is not a 'practical requirement' to have this design freedom to break the other symmetries. For example, a layered 3D PhC with an in-plane square profile, such that it has x, y, and diagonal mirror symmetries, can exhibit numerous symmetry band gaps along various high-symmetry planes (see updated supplemental). The updated supplemental also provides an example of a two-pillar design with only M_y symmetry that has a symmetry band gap. Anecdotally, we have generally found that similar layered 3D PhC structures exhibit such symmetry band gaps at normalized frequencies $f \sim 0.7(c/a)$, especially if the fill fraction and relative layer thicknesses can be loosely adjusted.

As to the true generality of symmetry band gaps in arbitrary structures with an appropriate symmetry, there are no general proofs of existence, nor are there no-go theorems. We are planning future studies to try to address this, but those would clearly be beyond the scope (and timeline) of the present manuscript. However, to address the reader's natural question regarding the generality of this behavior and the role of symmetry in the design paradigm, we have added further clarification and additional illustrative examples in the revised Supplementary Information.

Revisions made:

- We added Supplementary Fig. S2 and the corresponding discussion illustrating single- and multiple-symmetry design cases.

(Supplementary Information: Line 58 on page 1)
A second motif illustrates how multiple spatial symmetries generate multiple symmetry band gaps. As shown

in Fig. S2**b**, the square motif has parameters $W = 0.45$, $F = 0.35$, $n_1 = 3.5$, and $n_2 = 1.5$. Because this structure possesses M_x, M_y, and diagonal mirror symmetries, symmetry-resolved band gaps can appear along multiple mirror-invariant lines, including **Γ**–**X** and **Γ**–**M**. The corresponding five-period reflectance spectra show high reflectance within each symmetry band gap, confirming that the electromagnetic scattering response follows the symmetry-resolved band structure.

((New supplemental figure S2 for showing additional motif designs))

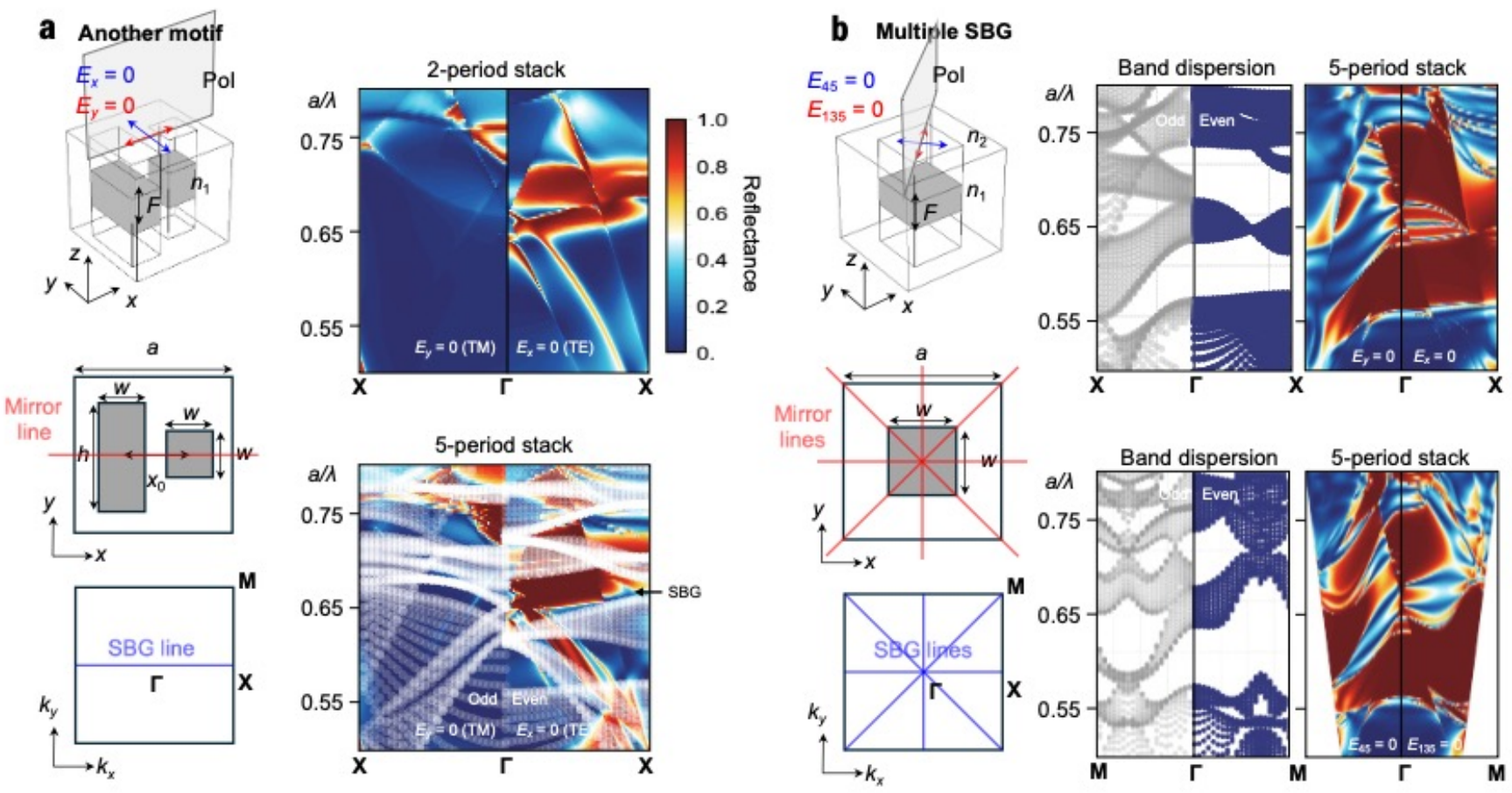

FIG. S2. **Another designs supporting symmetry band gaps in 3D photonic crystals.** **a**, Example of a unit cell possessing a single mirror symmetry. The left schematics show the real-space motif with the plane of incidence (PoI) for orthogonal polarization incidences and the corresponding symmetry band gap line in momentum space. The right panels show the reflectance spectra of finite stacks composed of the unit cell, including a two-period stack and a five-period stack overlaid with the symmetry-resolved projected band dispersion. The high-reflectance region appears only for the polarization channel that couples to the mirror-symmetry-forbidden sector, confirming the formation of a symmetry band gap along the corresponding mirror-invariant line. **b**, Example of multiple symmetry band gaps generated by a square motif with four mirror lines. Because the structure possesses multiple mirror symmetries in real space, symmetry band gaps are formed along the corresponding mirror-invariant lines in momentum space. The projected band dispersions and reflectance spectra are shown along both the **Γ**–**X** and **Γ**–**M** directions. In each case, the absence of symmetry-allowed bulk modes in the projected band structure correlates with high reflectance in the finite five-period stack, demonstrating that the electromagnetic scattering response directly reflects the underlying symmetry band gaps.

4. The agreement between simulation and experiment is quite good, in my view, for the high symmetry plane of interest (figure 3). While still quite reasonable, it appears notably less good along the θ_y direction (figure 4), especially some odd disconnects right at $\theta_y = 0$ compared to the θ_x plane. This is minor, and does not impact the evidence / claims of the main paper. However, some explanation/discussion as to this discrepancy in agreement may be appropriate.

(AR-B4) We thank the reviewer for this careful observation. We agree that, ideally, the spectra at $\theta_y = 0$ should be continuous between the θ_x and θ_y scans. In our original measurement configuration, however, these two scans were not performed under perfectly identical conditions: switching between them required mechanical rotation of the sample and polarizer, which could slightly change the alignment and the focal spot position on the patterned area. These factors likely caused the small discrepancy near $\theta_y = 0$.

To address this issue, we performed additional back-focal-plane imaging measurements in the revision. This measurement directly captures the isofrequency angular response without mechanically sweeping θ_x and θ_y, and confirms the angular selectivity of the device.

Revisions made:

- We added back-focal-plane imaging results in the main text and Supplementary Information.

(Updated figure 4 showing the isofrequency angular response without mechanical realignment)
(Main Text: Line 281 on page 6)

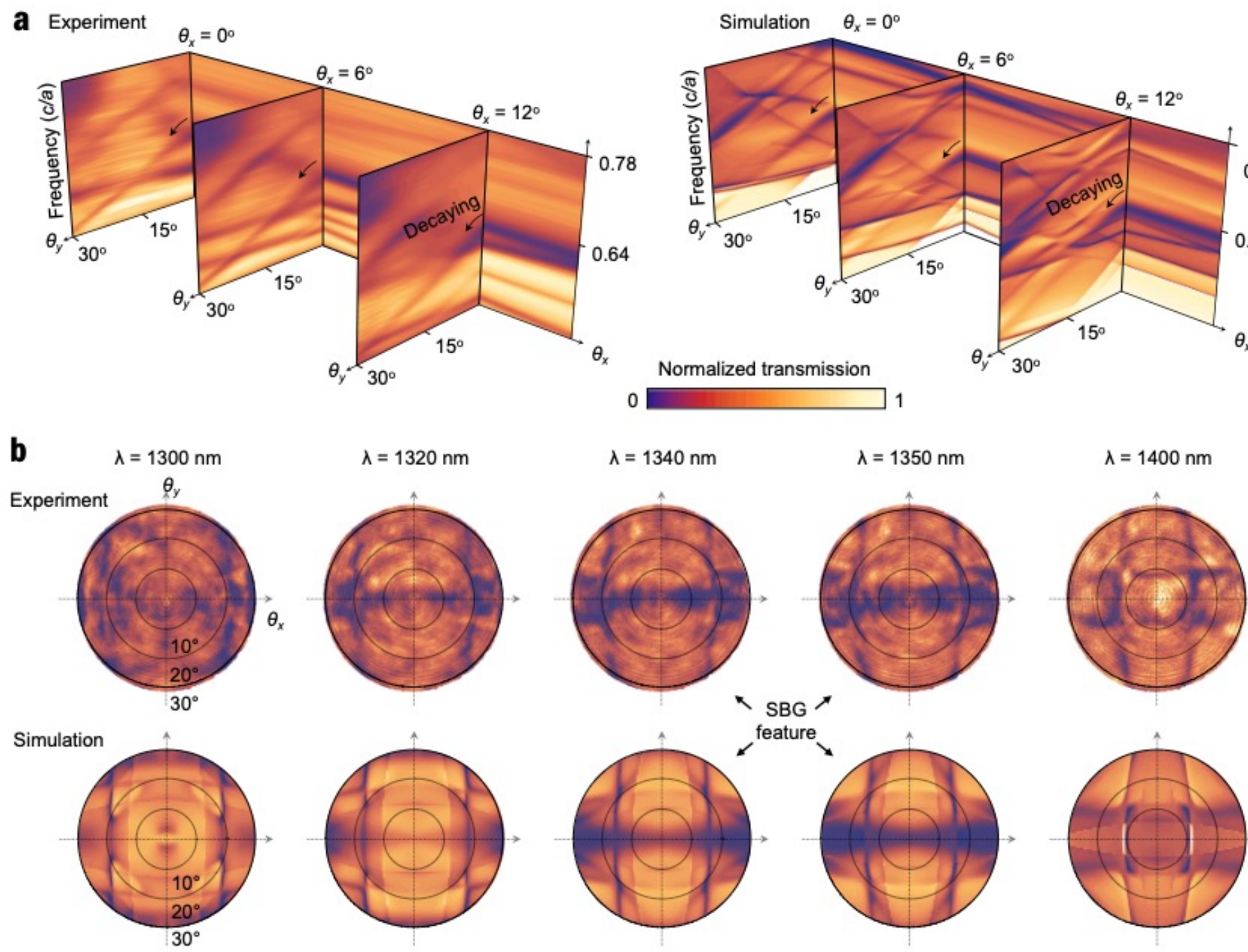

FIG. 4. **Experimental demonstration of intrinsic angular selectivity of the layered prism structure. a**, Substrate-normalized transmission polarized in $E_x = 0$ as a function of θ_y, obtained by rotating the incident angle away from a high-symmetry orientation for fixed $\theta_x = 0°$, $6°$, and $12°$. The transmission is strongly suppressed only near $\theta_y = 0°$, corresponding to alignment with the symmetry-defined incident plane, and rapidly recovers as the incident angle deviates from this direction. On the right side, corresponding numerical simulations of the polarization-dependent substrate-normalized transmission confirming that the angular selectivity arises from the layered prism structure's symmetry band gap. **b**, Normalized back-focal-plane transmission maps obtained experimentally (top row) and numerically (bottom row) for $E_x = 0$ incident polarization at representative wavelengths from $\lambda = 1300$ to 1400 nm, showing the symmetry band gap feature within the measured spectral range.

In addition, we perform transmission-mode back-focal-plane imaging to directly visualize the wavelength-dependent two-dimensional angular transmission response (see Supplementary Information Sec.SIII for details of the setup). The wavelength dependent k-space measurements further confirm the symmetry defined angular selectivity of the TE transmission suppression, see Fig.4**b**. At wavelengths inside the symmetry band gap, $\lambda = 1340$ and 1350 nm, the transmission is strongly suppressed along the $\theta_y = 0$ symmetry condition and rapidly recovers away from this plane. By contrast, the suppression disappears at off-symmetry band gap wavelengths, $\lambda = 1300$, 1320, and 1400 nm. These back-focal-plane images provide a direct two-dimensional visualization of the symmetry band gap and confirm that the TE suppression is spectrally confined to the symmetry band gap region.

(Supplementary Information: Line 131 on page 2)

To directly map the two-dimensional angular transmission response, transmission-mode k-space imaging measurements were performed using a back-focal-plane imaging setup, as schematically illustrated in Fig.S5. A broadband super continuum source (NKT Photonics SuperK Compact) was used as the illumination source for the k-space transmission measurements. The incident polarization was controlled using a broadband linear polarizer operating in the wavelength range of 1100–1800 nm. For wavelength-selective k-space imaging, a hard-coated bandpass filters were inserted in the imaging path. Bandpass filters centered at 1300, 1320, 1340, 1350, and 1400 nm, each with a full width at half maximum of 12 nm, were used to acquire k-space transmission maps at the corresponding wavelengths (Throlabs FBH1300-12, FBH1320-12, FBH1340-12, FBH1350-12, and FBH1400-12). The incident light was focused onto the sample using an objective lens (L1, Olympus LCPlanFL 40×, NA = 0.60). The transmitted light was collected by a NIR objective lens (L2,

Olympus LCPLN50× NIR, NA = 0.65) placed on the opposite side of the sample. The back focal plane of the collection objective was relayed onto the k-space imaging camera using a 4f relay system composed of two lenses with focal lengths of 200 mm (L3, Thorlabs AC254-200-C) and 150 mm (L4, Thorlabs AC254-150-C). The k-space images were recorded using a VSWIR camera (Alvium 1800 U-130 VSWIR). Since each position in the back-focal-plane image corresponds to a transmitted wavevector component, this configuration enables two-dimensional mapping of the transmission response along both θ_x and θ_y. A separate real-space imaging path was used to align the patterned region of the sample with the illumination spot prior to k-space image acquisition. For real-space imaging, the sample was illuminated using a white-light lamp (Thorlabs SLS201L), and the real-space image was recorded using a NIR camera (CS135MUN NIR). The real-space imaging path allowed accurate positioning of the patterned region before acquiring the k-space transmission images. All k-space transmission images were normalized by reference images obtained from an unpatterned region of the same substrate under identical illumination, polarization, wavelength-filtering, and imaging conditions. This normalization accounts for the system response, substrate transmission, and angle-dependent collection efficiency.

(Supplemental Figure for back-focal imaging setup)

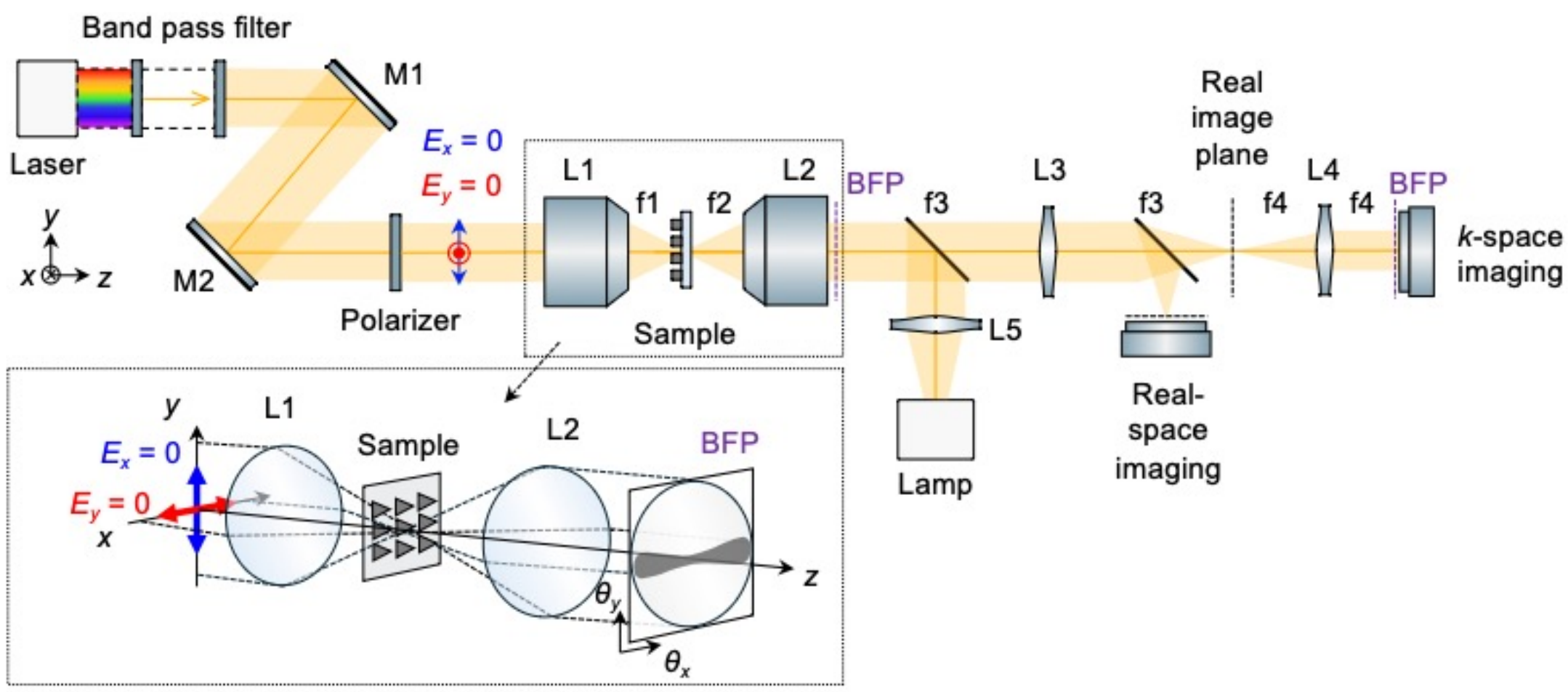

FIG. S5. **Transmission-mode back-focal-plane imaging setup based on a 4f relay system.** Schematic of the back-focal-plane (BFP) imaging setup. The beam path was folded by two mirrors (M1 and M2) while maintaining a parallel optical axis through the system. The incident polarization was controlled as TE or TM using a linear polarizer. The incident light was focused onto the sample by the objective lens L1, and the transmitted light was collected by the objective lens L2. The BFP of L2 was relayed onto the k-space imaging camera using relay lenses L3 and L4. The enlarged view highlights the imaging relation around the sample, where the real-space sample plane and the Fourier-space BFP plane are shown on opposite sides of the objective pair. At the BFP of L2, the transmitted angular distribution is encoded as a k-space intensity pattern, with positions in the BFP corresponding to the angular coordinates θ_x and θ_y in momentum space. A separate real-space imaging path was implemented using a white-light lamp and a lens (L5, $f = 75$ mm) to provide Köhler illumination for sample alignment.

5. I agree with the point in the conclusion of the potential practical advantages of this being a non-resonant effect. Some mention earlier may also be appropriate, as it is a distinguishing aspect of this work and the idea of a symmetry bandgap. However, I would also be curious if such an effect (over some resonant bandwidth) is expected to be feasible with resonant devices? I notice the authors have this related expertise, and hence may comment. For instance, the "flat band" device in Ref. [R1] seems to have the potential quite

comparable in behavior, but with a distinct mechanism (and much narrower bandwidth). Or, is there some fundamental barrier to achieving something like this with resonant features with low Q-factor to make it more comparable to the 20nm bandwidth observed here? Some more comparison/discussion, and especially and explicit mention of whether the equivalent is expected to be achievable with a resonant mechanisms (albeit less practically) would be helpful. [R1] H.S. Nguyen, et al. "Symmetry Breaking in Photonic Crystals: On-Demand Dispersion from Flatband to Dirac Cones", Physical Review Letters 120, 066102, (2018).

(AR-B5) We thank the reviewer for this helpful suggestion. We agree that the non-resonant nature of the symmetry-band-gap response is a distinguishing aspect of our work, and we have revised the manuscript to mention this point earlier in the text.

We also agree that resonant photonic structures can, in principle, produce angularly selective optical responses over a finite bandwidth. For example, the flat-band photonic-crystal structures discussed in Ref. [R1] provide an important comparison point because engineered resonant dispersion can generate reflection or transmission features that are extended along selected directions in momentum space. Such devices may therefore be able to reproduce some aspects of the angular response demonstrated here, particularly over a narrow spectral range.

However, resonant implementations face a general tradeoff between operational bandwidth and angular selectivity. In a resonant angular filter, the response is governed by the resonant frequency $\omega_0(\mathbf{k}_\parallel)$ and the resonance linewidth γ. Increasing the bandwidth requires increasing γ, or equivalently reducing the quality factor. But sharp angular selectivity requires the resonance frequency to move away from the operating frequency rapidly as the incident wavevector is displaced in the rejected angular direction. If the linewidth is broadened while the angular dispersion remains modest, then a larger range of incident angles remains within the resonance linewidth, reducing the angular selectivity. Conversely, maintaining narrow angular selectivity generally requires either a relatively high-Q resonance or very strong angular dispersion in the rejected direction. Thus, while low-Q resonant devices may be made broader-band, doing so tends to make it more difficult to preserve the narrow transverse angular response needed here.

Of course, this trade-off in resonant structures is avoided in our non-resonant approach, where the bandwidth of our device is set by the symmetry band gap rather than by the linewidth of an individual resonance, allowing broadband angular selectivity without relying on a low-Q resonant feature.

Revisions made:

- We have added a mention of engineered angular responses in photonic-crystal-based approaches in the Introduction. (Note, we have kept the manuscript focused on the symmetry-band-gap mechanism in a 3D photonic crystal, rather than expanding the discussion to resonant angular-selective devices, since the latter represents a distinct design paradigm outside the central scope of the present work.)

- We have also added a few other earlier mentions of the non-resonant nature of our device.

(Main Text: Line 45 on page 1)
While prior metasurface, metamaterial, and photonic crystal–based approaches have demonstrated individual aspects of these capabilities, including wide-field polarization control, engineered angular responses, and compact analog image processing [38–52],...

(Added reference:

[43] H. S. Nguyen, F. Dubois, T. Deschamps, S. Cueff, A. Pardon, J.-L. Leclercq, C. Seassal, X. Letartre, and P. Viktorovitch, Symmetry Breaking in Photonic Crystals: On-Demand Dispersion from Flatband to Dirac Cones, Phys. Rev. Lett. 120, 066102 (2018)

Reviewer C

The manuscript proposes a truncated three-dimensional photonic crystal that exhibits a symmetry band gap along a high-symmetry direction, and experimentally demonstrates its operation as a compact, all-dielectric optical element capable of simultaneous polarization separation and angular selectivity. The experimental results are generally consistent with the theoretical design. In particular, polarization-dependent angular selectivity has, in my view, already been demonstrated in previous works, including Science 343, 1499 (2014), ACS Photonics 5, 4125 (2018), and Nature Photonics 7, 791 (2013). More broadly, it is well known that anisotropic structures, or structures under oblique illumination, can exhibit polarization-dependent spectral responses. Therefore, the statement in the abstract that this work "extends polarization control into the angular domain" appears somewhat misleading or at least overstated. On the other hand, although the polarization-dependent angular spectrum here may differ from prior works, I can not find the unique value in terms of applications and I am concerned about its broad interest in the society. Considering the high-quality of Nature Communications, I can not recommend for the publication.

(AR-C0) We thank the referee for their efforts in helping us to improve our study.

We agree that polarization-dependent angular spectra can occur in anisotropic structures and under oblique illumination, and we did not intend to claim that polarization-dependent angular selectivity is itself unprecedented. Rather, the central distinction of our work is that the angular and polarization selectivity arise from a symmetry band gap of a 3D photonic crystal. Along the mirror-symmetric incidence direction, one incident polarization is symmetry-forbidden from coupling to propagating modes in the PhC within the symmetry band gap, resulting in suppressed transmission, whereas the orthogonal polarization remains transmissive. When the incident wavevector is moved away from the symmetry-defined direction, the mirror constraint is lifted and the polarization-selective response disappears. Thus, the polarization dependence is not a conventional oblique-incidence or anisotropic-material effect, but is tied directly to the symmetry structure of the photonic band diagram. This distinction means that our device exhibits a polarization-selective transmission/reflection response over an extended incidence plane. To the best of our knowledge, this form of line-like polarization-selective angular response has not been predicted or demonstrated in prior compact angle-selective optical systems.

To avoid any possible ambiguity, we have revised the wording in the abstract, please see the updated abstract.

We also thank the reviewer for bringing these important references to our attention, and agree that these prior works are relevant to the broader context of angle-selective optical filtering and provide useful points of comparison for our study. At the same time, we wish to emphasize that our work is clearly differentiated from the cited studies in terms of both the physical origin of the angular selectivity and the experimental implementation. We clarify these differences below.

- Science **343**, 1499 (2014) and ACS Photonics **5**, 4125 (2018)

The Science work is an important demonstration of broadband angle-selective transmission using a multilayer stack consisting essentially of a series of slightly different Bragg stacks. In that study, angle and polarization selectivity is achieved by relying on the Brewster angle of the dielectric materials used in the Bragg stacks, and then varying the layer thicknesses of each constituent Bragg stack. Overall, this yields transmission for a single incident angle for p-polarized light, corresponding to the Brewster angle and corresponding incident polarization, with all other incident light being reflected due to the stop bands of the Bragg stacks. ACS Photonics then extends this class of 1D multilayer angular filters toward polarization-independent operation by combining anisotropic 1D PhC stacks with half-wave-plate layers.

Our work is distinct from these prior studies, both in how it achieves its angular selectivity (via a 3D PhC vs a 1D PhC) and in the nature of the transmitted angular range (our system enables a line in (θ_x, θ_y) of high reflectivity vs a single point of high transmission). Moreover, it is not clear to us that the Brewster-angle physics used in these prior studies can be directly generalized to produce this line-like angular response. As

such, our work demonstrates a distinct route to broadband angular and polarization selectivity.

- Nature Photonics **7**, 791 (2013).

This prior study demonstrated an all-dielectric zero-index metamaterial in which angular selectivity is associated with near-zero-index behavior near a Dirac-cone condition. This approach is also distinct from our mechanism for achieving a selective optical response. In that case, the angular response is tied to resonantly engineered zero-index dispersion near the Dirac-point frequency. In our structure, the response originates from a non-resonant symmetry band gap of a 3D PhC, allowing the angular and polarization selectivity to be understood directly from the separation of mirror-parity sectors along the high-symmetry line.

Thus, while Fig. 4 of that work shows angularly selective transmission near the zero-index condition, the response is tied to operation near the Dirac-point frequency. By contrast, our use of a 3D photonic crystal to generate a symmetry band gap yields narrow angular selectivity in the transverse direction, approximately $\pm 5°$, while maintaining the selective response over a broader range of k_x and ω. Our experimental device realizes these distinguishing features over a wavelength range of approximately 20nm, and the same symmetry-band-gap mechanism can be further broadened by using larger-index-contrast materials. For example, in an Si/SiO_2 structure, our approach could yield $\leq \pm 5°$ angular selectivity (perpendicular to the high-symmetry plane of incidence) over a bandwidth of $\geq$ 50nm.

Altogether, we agree that the cited works provide valuable comparison points, while emphasizing that our system's symmetry-band-gap origin, line-like momentum-space response, and compact 3D PhC implementation distinguish it from these prior demonstrations.

To address this comment, we have added citations to the mentioned prior works and amended the relevant sections of the abstract and introduction to better situate our work within the context of prior studies. (See also the response below, AR-C1.)

Other comments are following:

(1) The authors are supposed to discuss more thoroughly recent progresses on simultaneous polarization separation and angular selectivity in the introduction. In particular, the authors should provide a more detailed comparison with prior studies for claiming their novelty in design or applications.

(AR-C1) We appreciate the referee's desire for a thorough review of the prior literature in the introduction, and we have expanded the relevant discussion therein, including the references identified above and by the other referees, as well as other references we have subsequently identified.

Revisions made:

- We revised the second paragraph in the introduction to provide a more thorough discussion of prior work.

(Main Text: Line 45 on page 1)
While prior metasurface, metamaterial, and photonic crystal–based approaches have demonstrated individual aspects of these capabilities, including wide-field polarization control, engineered angular responses, and compact analog image processing [38–52],...

(Added references are listed below:

[38] H. Jiang, H. Chen, H. Li, Y. Zhang, and S. Zhu, Omni-directional gap and defect mode of one-dimensional photonic crystals containing negative-index materials, Applied Physics Letters 83, 5386 (2003)

[39] P. Moitra, Y. Yang, Z. Anderson, I. I. Kravchenko, D. P. Briggs, and J. Valentine, Realization of an all-dielectric zero-index optical metamaterial, Nature Photon. 7, 791 (2013)

[40] Y. Shen, D. Ye, I. Celanovic, S. G. Johnson, J. D. Joannopoulos, and M. Soljačić, Optical Broadband Angular Selectivity, Science 343, 1499 (2014)

[42] Y. Qu, Y. Shen, K. Yin, Y. Yang, Q. Li, M. Qiu, and M. Soljačić, Polarization-Independent Optical Broad band Angular Selectivity, ACS Photonics 5, 4125 (2018).

[44] F. Wu, G. Lu, C. Xue, H. Jiang, Z. Guo, M. Zheng,C. Chen, G. Du, and H. Chen, Experimental demonstration of angle-independent gaps in one-dimensional photonic crystals containing layered hyperbolic metamaterials and dielectrics at visible wavelengths, Applied Physics Letters 112, 041902 (2018)

[45] F. Wu, G. Lu, Z. Guo, H. Jiang, C. Xue, M. Zheng, C. Chen, G. Du, and H. Chen, Redshift gaps in one-dimensional photonic crystals containing hyperbolic metamaterials, Phys. Rev. Appl. 10, 064022 (2018))

(2) The authors claim that a symmetry band gap will lead to high reflection and a disappeared band gap will allow the transmission. However, even in the small-angle region where a cluster of odd modes exists and the infinite crystal no longer appears to have a clear band gap, the two-period structure still show high reflection. The relationship between high reflection and the presence or absence of the symmetry band gap therefore needs to be explained more carefully.

(AR-C2) We thank the reviewer for pointing out the need to clarify the relationship between the symmetry band gap and the observed high-reflection response. The full 3D PhC does not possess a complete photonic band gap. However, along the $k_y = 0$ high-symmetry line, the mirror symmetry M_y separates the Bloch modes into even and odd symmetry sectors. In this symmetry-resolved band structure, a frequency range can exist where the even-in-E_y modes are absent while odd-in-E_y modes remain available. We refer to this absence of one symmetry sector as a symmetry band gap.

For incident light aligned with the high-symmetry plane, the $E_x = 0$ channel, corresponding to TE or s polarization, has even-in-E_y symmetry. Therefore, for frequencies within the symmetry band gap, it has no symmetry-matched propagating mode to couple to inside the PhC, and its transmission is suppressed. By contrast, the $E_y = 0$ channel, corresponding to TM or p polarization, can couple to the available odd-in-E_y modes and is therefore symmetry-allowed to transmit through the PhC.

Importantly, however, "symmetry-allowed transmission" does not mean that the TM channel must be perfectly transmitted. It only means that transmission is not forbidden by the symmetry band gap. In a finite-thickness structure, additional optical effects, such as guided-mode resonances or thin-film interference, can still produce reflection features in the symmetry-allowed channel. We believe that the high-reflection features observed near the Γ point for the TM channel in the two-period triangular-prism structure arise from such finite-structure resonant effects, rather than from the symmetry band gap itself.

To make this distinction clearer, we have added a new example in the revised Supplementary Information. This additional motif also supports a symmetry band gap and produces strong reflection for the symmetry-forbidden TE channel, while the symmetry-allowed TM channel remains largely transmissive without the pronounced reflection features seen in the triangular-prism motif. This comparison confirms that the symmetry band gap explains the suppressed transmission of the forbidden channel, whereas extra reflection features in the allowed channel are motif-dependent finite-structure effects.

Revisions made:

- We added a Supplementary figure S2 (a) showing an additional motif in which the symmetry-forbidden channel is strongly reflected while the symmetry-allowed channel remains largely transmissive.
- We clarified that symmetry-allowed transmission means that transmission is not forbidden by symmetry, but does not preclude additional reflection caused by finite-structure resonances or thin-film effects.

(Supplementary Information: Line 43 on page 1)

To demonstrate the generality of the proposed symmetry-based mechanism, we consider additional 3D PhC motifs supporting symmetry band gaps. As shown in Fig. S2(a), the first motif consists of rectangular and square blocks with refractive indices n_1 and n_2, surrounded by air. The structural parameters are $F = 0.25$, $W = 0.3$, $h = 0.6$, $x_0 = 0.5$, $n_1 = 3.5$, and $n_2 = 1.5$. This motif preserves a single mirror symmetry M_y, producing a symmetry band gap along the corresponding $\mathbf{\Gamma}$–$\mathbf{X}$ line. The calculated reflectance spectra for two- and five-period stacks show high reflectance for the $E_x = 0$ polarization channel while the symmetry-allowed $E_y = 0$ channel remains largely transmissive, consistent with the absence of symmetry-matched propagating modes in the corresponding symmetry sector. A second motif illustrates how multiple spatial symmetries generate multiple symmetry band gaps. As shown in Fig. S2(b), the square motif has parameters $W = 0.45$, $F = 0.35$, $n_1 = 3.5$, and $n_2 = 1.5$. Because this structure possesses M_x, M_y, and diagonal mirror symmetries, symmetry-resolved band gaps can appear along multiple mirror-invariant lines, including $\mathbf{\Gamma}$–$\mathbf{X}$ and $\mathbf{\Gamma}$–$\mathbf{M}$. The corresponding five-period reflectance spectra show high reflectance within each symmetry band gap, confirming that the electromagnetic scattering response follows the symmetry-resolved band structure.

(Newly added Supplementary figure showing that the symmetry-allowed channel can remain largely transmissive in an alternative motif)

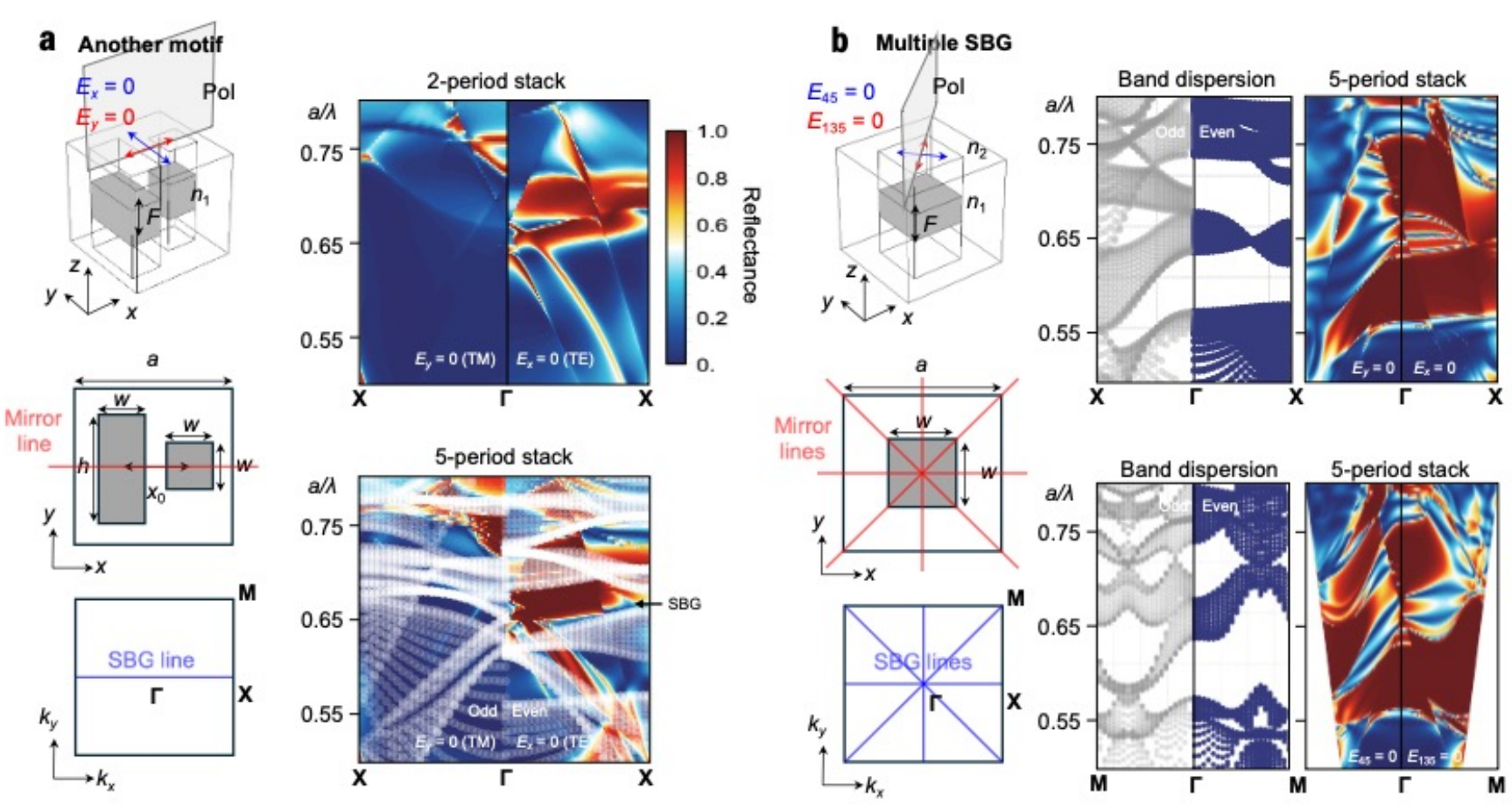

FIG. S2. **Another designs supporting symmetry band gaps in 3D photonic crystals.** **a**, Example of a unit cell possessing a single mirror symmetry. The left schematics show the real-space motif with the plane of incidence (PoI) for orthogonal polarization incidences and the corresponding symmetry band gap line in momentum space. The right panels show the reflectance spectra of finite stacks composed of the unit cell, including a two-period stack and a five-period stack overlaid with the symmetry-resolved projected band dispersion. The high-reflectance region appears only for the polarization channel that couples to the mirror-symmetry-forbidden sector, confirming the formation of a symmetry band gap along the corresponding mirror-invariant line. **b**, Example of multiple symmetry band gaps generated by a square motif with four mirror lines. Because the structure possesses multiple mirror symmetries in real space, symmetry band gaps are formed along the corresponding mirror-invariant lines in momentum space. The projected band dispersions and reflectance spectra are shown along both the $\mathbf{\Gamma}$–$\mathbf{X}$ and $\mathbf{\Gamma}$–$\mathbf{M}$ directions. In each case, the absence of symmetry-allowed bulk modes in the projected band structure correlates with high reflectance in the finite five-period stack, demonstrating that the electromagnetic scattering response directly reflects the underlying symmetry band gaps.

(3) In experiment, the most common polarization bases are linear polarization, such as s/p or x/y polarization, and circular polarization. By contrast, the polarization condition defined by Ex=0 when θ_x and θ_y are nonzero, as used in fig. 2b, appears rather specific and may be of limited practical relevance.

(AR-C3) We thank the reviewer for raising this important point. Since our manuscript describes the interaction between symmetry-classified PhC modes and externally incident polarization states, a consistent definition of the incident polarization basis is essential for avoiding confusion, and our choice of basis is

motivated by the desire to simplify the connection to the modes of the 3D PhC. However, we note that for $\theta_y = 0$ incident light, the $E_x = 0$ and $E_y = 0$ polarization basis that we use in the manuscript are precisely s- and p-polarized light, respectively. Off of $\theta_y = 0$, this choice of basis still spans polarization space, confirming that the effect we are observing is quite general.

Revisions made:

- We revised the beginning of the Results section to define the incident polarization notation more rigorously and used this notation consistently throughout the manuscript.

(Main Text: Line 141 on page 3)
For frequencies within the symmetry band gap, only odd-in-E_y modes are available along the $\mathbf{\Gamma}$–$\mathbf{X}$ high-symmetry line. Therefore, any even-in-E_y incident light has a vanishing symmetry overlap with these odd-in-E_y modes and cannot couple to a propagating mode inside the PhC, resulting in suppressed transmission. For incoming light with a wavevector in the xz-plane, the polarization condition $E_x = 0$ corresponds to an incident field with only an even-in-E_y electric-field component, independent of θ_x (see Fig. 1**a**), as the field profile of the incoming plane wave has no dependence on y. Note that for $k_y = 0$, the $E_x = 0$ polarization is the transverse-electric (TE), or s-polarized, free-space channel. Thus, TE incident light is expected to be suppressed within the symmetry band gap. In contrast, transverse-magnetic (TM), or p-polarized, incident light with $\theta_y = 0$ satisfies $E_y = 0$, which corresponds to non-zero E_{xz} and is not symmetry-forbidden from coupling to the available odd-in-E_y modes, allowing transmission through the PhC. When the incident light is not aligned with the high-symmetry line, $\theta_y \neq 0$, the mirror symmetry constraint is lifted, and both incident polarizations can couple to the modes of the 3D PhC. In the following, we use the free-space polarization basis $E_x = 0$ and $E_y = 0$ to denote the two incident polarization channels for arbitrary incident angles (θ_x, θ_y), which maintains a straightforward connection to the separable symmetry sectors of the layered prism 3D PhC when $\theta_y = 0$.

(4) Although Fig. 4 shows an angle-dependent spectrum, I am not fully convinced that it demonstrates practically useful angular selectivity, because other resonant features are also present in the wavelength range of interest and may introduce crosstalk. What's more, to better show the angular selectivity, the authors are supposed to present a series of two-dimension reflection in momentum space under different incident wavelengths.

(AR-C4) We appreciate the referee's desire to see a back focal plane measurement of our system to confirm its simultaneous angular and polarization selectivity. We agree with this suggestion and we have performed back focal plane transmission experiments at a series of different wavelengths both inside and outside the symmetry band gap's spectral extent. These momentum-space measurements show that the suppressed-transmission feature associated with the symmetry band gap is localized near the $k_y = 0$ high-symmetry direction and decays rapidly as the incident wavevector deviates from this direction, confirming our prior claims of our device's selectivity.

Regarding the question of "crosstalk." We interpret the concern about crosstalk as referring to the possibility that additional features in the 3D PhC's spectrum near the symmetry-band-gap response could produce unwanted transmission or reflection responses, thereby reducing the angular or polarization selectivity of the device. In our back focal plane measurements for frequencies within the symmetry band gap, we do not observe additional momentum-space features that produce comparable suppressed-transmission channels away from the intended angular region. Thus, the revised Fig. 4b directly demonstrates that the angular selectivity is maintained in two-dimensional momentum space and is not substantially degraded by nearby optical features. In other words, we do not observe, either in simulation or experiment, any crosstalk. More broadly, given the large design space available to find symmetry band gaps, as all that is needed is a single reflection symmetry, it should generically be possible to design such layered 3D PhC systems that do not exhibit any deleterious effects of crosstalk when used as a angle- and polarization-selective reflector.

Revisions made:

- We added back-focal-plane imaging results in the main text and Supplementary Information.

(Updated figure 4 including back-focal plane measurements)

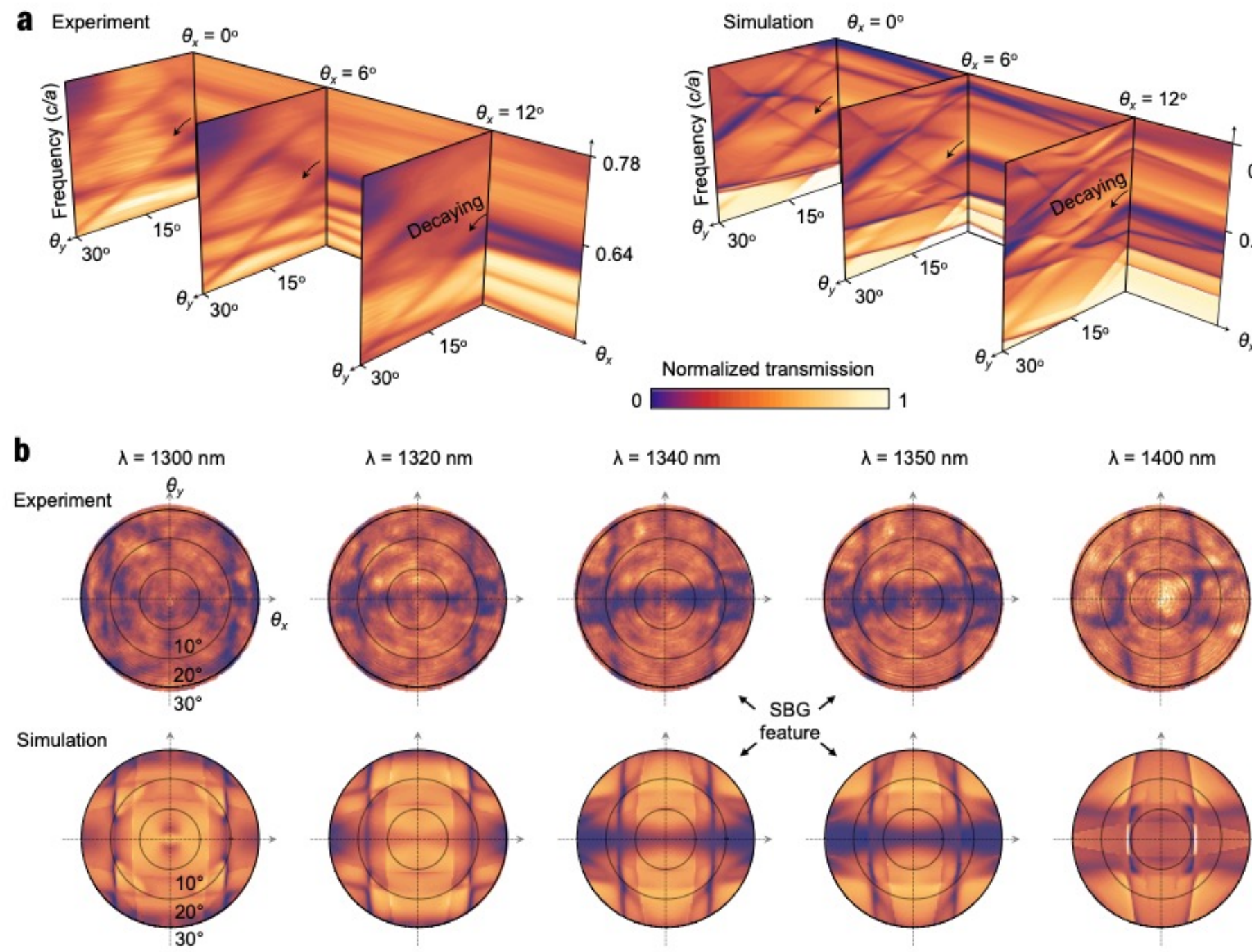

FIG. 4. **Experimental demonstration of intrinsic angular selectivity of the layered prism structure. a**, Substrate-normalized transmission polarized in $E_x = 0$ as a function of θ_y, obtained by rotating the incident angle away from a high-symmetry orientation for fixed $\theta_x = 0°$, $6°$, and $12°$. The transmission is strongly suppressed only near $\theta_y = 0°$, corresponding to alignment with the symmetry-defined incident plane, and rapidly recovers as the incident angle deviates from this direction. On the right side, corresponding numerical simulations of the polarization-dependent substrate-normalized transmission confirming that the angular selectivity arises from the layered prism structure's symmetry band gap. **b**, Normalized back-focal-plane transmission maps obtained experimentally (top row) and numerically (bottom row) for $E_x = 0$ incident polarization at representative wavelengths from $\lambda = 1300$ to 1400 nm, showing the symmetry band gap feature within the measured spectral range.

(Main Text: Line 281 on page 6)
In addition, we perform transmission-mode back-focal-plane imaging to directly visualize the wavelength-dependent two-dimensional angular transmission response (see Supplementary Information Sec.SIII for details of the setup). The wavelength dependent k-space measurements further confirm the symmetry defined angular selectivity of the TE transmission suppression, see Fig.4**b**. At wavelengths inside the symmetry band gap, $\lambda = 1340$ and 1350 nm, the transmission is strongly suppressed along the $\theta_y = 0$ symmetry condition and rapidly recovers away from this plane. By contrast, the suppression disappears at off-symmetry band gap wavelengths, $\lambda = 1300$, 1320, and 1400 nm. These back-focal-plane images provide a direct two-dimensional visualization of the symmetry band gap and confirm that the TE suppression is spectrally confined to the symmetry band gap region.

(Supplemental Figure for back-focal imaging setup)

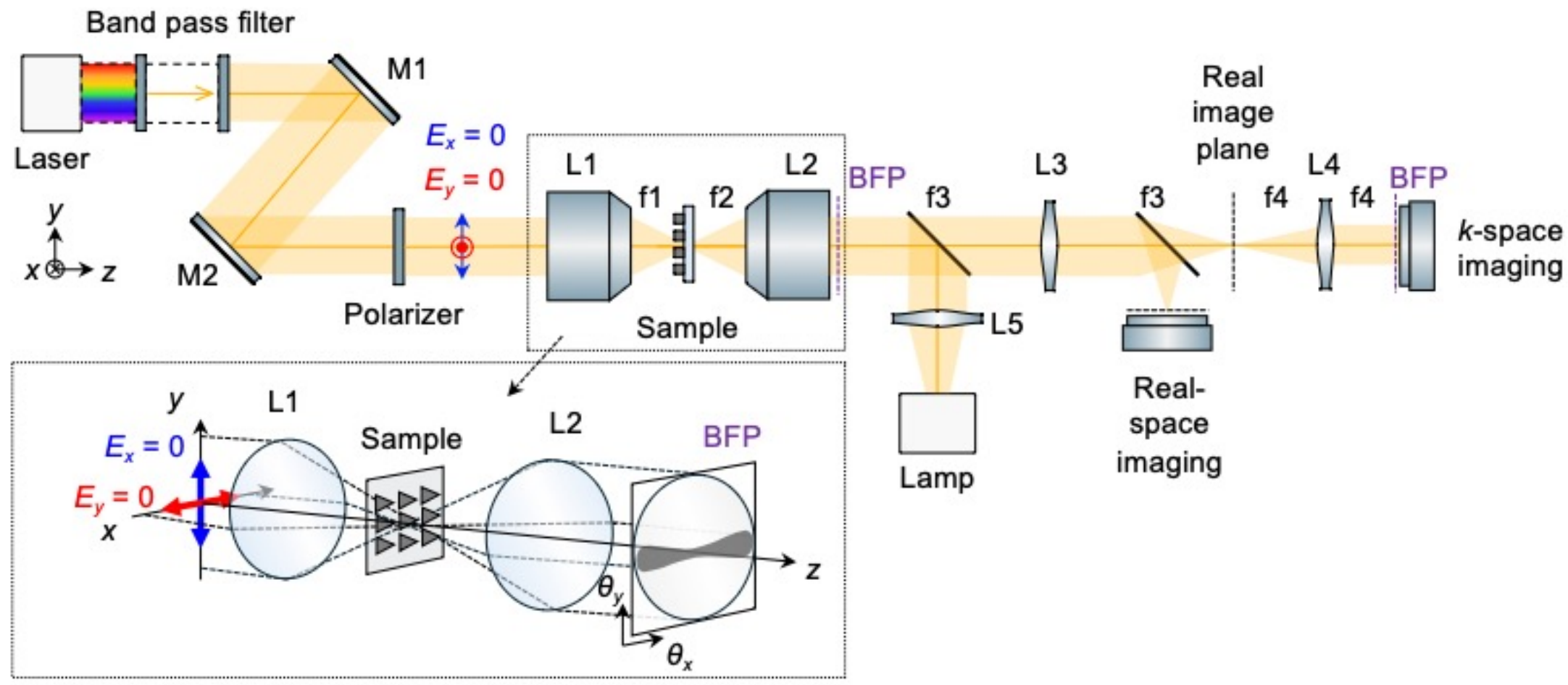

FIG. S5. **Transmission-mode back-focal-plane imaging setup based on a 4f relay system.** Schematic of the back-focal-plane (BFP) imaging setup. The beam path was folded by two mirrors (M1 and M2) while maintaining a parallel optical axis through the system. The incident polarization was controlled as TE or TM using a linear polarizer. The incident light was focused onto the sample by the objective lens L1, and the transmitted light was collected by the objective lens L2. The BFP of L2 was relayed onto the k-space imaging camera using relay lenses L3 and L4. The enlarged view highlights the imaging relation around the sample, where the real-space sample plane and the Fourier-space BFP plane are shown on opposite sides of the objective pair. At the BFP of L2, the transmitted angular distribution is encoded as a k-space intensity pattern, with positions in the BFP corresponding to the angular coordinates θ_x and θ_y in momentum space. A separate real-space imaging path was implemented using a white-light lamp and a lens (L5, $f = 75$ mm) to provide Köhler illumination for sample alignment.

(Supplementary Information: Line 131 on page 2)

To directly map the two-dimensional angular transmission response, transmission-mode k-space imaging measurements were performed using a back-focal-plane imaging setup, as schematically illustrated in Fig.S5. A broadband super continuum source (NKT Photonics SuperK Compact) was used as the illumination source for the k-space transmission measurements. The incident polarization was controlled using a broadband linear polarizer operating in the wavelength range of 1100–1800 nm. For wavelength-selective k-space imaging, a hard-coated bandpass filters were inserted in the imaging path. Bandpass filters centered at 1300, 1320, 1340, 1350, and 1400 nm, each with a full width at half maximum of 12 nm, were used to acquire k-space transmission maps at the corresponding wavelengths (Throlabs FBH1300-12, FBH1320-12, FBH1340-12, FBH1350-12, and FBH1400-12). The incident light was focused onto the sample using an objective lens (L1, Olympus LCPlanFL 40×, NA = 0.60). The transmitted light was collected by a NIR objective lens (L2, Olympus LCPLN50× NIR, NA = 0.65) placed on the opposite side of the sample. The back focal plane of the collection objective was relayed onto the k-space imaging camera using a 4f relay system composed of two lenses with focal lengths of 200 mm (L3, Thorlabs AC254-200-C) and 150 mm (L4, Thorlabs AC254-150-C). The k-space images were recorded using a VSWIR camera (Alvium 1800 U-130 VSWIR). Since each position in the back-focal-plane image corresponds to a transmitted wavevector component, this configuration enables two-dimensional mapping of the transmission response along both θ_x and θ_y. A separate real-space imaging path was used to align the patterned region of the sample with the illumination spot prior to k-space image acquisition. For real-space imaging, the sample was illuminated using a white-light lamp (Thorlabs SLS201L), and the real-space image was recorded using a NIR camera (CS135MUN NIR). The real-space imaging path allowed accurate positioning of the patterned region before acquiring the k-space transmission images. All

k-space transmission images were normalized by reference images obtained from an unpatterned region of the same substrate under identical illumination, polarization, wavelength-filtering, and imaging conditions. This normalization accounts for the system response, substrate transmission, and angle-dependent collection efficiency.

Comments

In the submitted manuscript, the authors theoretically and experimentally realized polarization-dependent angular selection in 3D photonic crystals (PhCs). When incident light is aligned along the high-symmetry direction, the symmetry photonic band gap (PBG) produces polarization-dependent reflection and transmission. By contrast, deviation from this orientation lifts the symmetry constraint, resulting in the disappearance of polarization selectivity. Overall, I think this manuscript is presented in a good shape in terms of sound theoretical foundations, rich visualizations, and rigorous validations. Nonetheless, the writing quality of the manuscript could be further improved. After the authors revise the manuscript based on the following comments, I would recommend the acceptance of this manuscript for publication in *Nature Communications*.

1) From the results in Fig. 2(a), the PBG for odd channel is distinct from that from even channel. Please provide the underlying physical reason.

2) Without loss of generality, I suggest the authors utilize pillars with alternative geometries (e.g., T-shaped pillars) in the SM to realize polarization-dependent angular selection.

3) In Fig. 2(a), the authors give the results for two periods. To better understand the transition from finite-period to infinite-period cases, the results for three and five periods should be given in the SM.

4) On page 2, the authors mention that "when the materials' index contrast is sufficiently large, $\Delta n \geq 0.8$". The authors need to clarify whether n_1 or n_2 is fixed.

5) Over the past two decades, researchers achieved angle-independent PBGs [Appl. Phys. Lett. **83**, 5386 (2003); Appl. Phys. Lett. **112**, 041902 (2018)] and redshift PBGs [Phys. Rev. Appl. **10**, 064022 (2018)] in 1D PhCs containing metamaterials. As the incident angle increases, the redshift PBGs shifts towards longer wavelengths. The authors could consider involving these works in the introduction section.

6) Other small issues:

- In Fig. 2(a), the values of 0.25 and −0.25 do not correspond to the midpoint between

points X and Γ.

- In Figs. 2(b) and 2(c), please add the unit of the incident angle.
